# Regulation of growth–defense balance by the JASMONATE ZIM‐DOMAIN (JAZ)‐MYC transcriptional module

**DOI:** 10.1111/nph.14638

**Published:** 2017-06-26

**Authors:** Ian T. Major, Yuki Yoshida, Marcelo L. Campos, George Kapali, Xiu‐Fang Xin, Koichi Sugimoto, Dalton de Oliveira Ferreira, Sheng Yang He, Gregg A. Howe

**Affiliations:** ^1^ Department of Energy‐Plant Research Laboratory Michigan State University East Lansing MI 48824 USA; ^2^ Department of Plant Biology Michigan State University East Lansing MI 48824 USA; ^3^ Howard Hughes Medical Institute Michigan State University East Lansing MI 48824 USA; ^4^ Plant Resilience Institute Michigan State University East Lansing MI 42284 USA; ^5^ Department of Biochemistry and Molecular Biology Michigan State University East Lansing MI 48824 USA

**Keywords:** gene cluster, glucosinolate, growth–defense tradeoffs, jasmonate (JA), plant defense, plant hormone, plant–insect interaction, triterpenoid

## Abstract

- The plant hormone jasmonate (JA) promotes the degradation of JASMONATE ZIM‐DOMAIN (JAZ) proteins to relieve repression on diverse transcription factors (TFs) that execute JA responses. However, little is known about how combinatorial complexity among JAZ–TF interactions maintains control over myriad aspects of growth, development, reproduction, and immunity.
- We used loss‐of‐function mutations to define epistatic interactions within the core JA signaling pathway and to investigate the contribution of MYC TFs to JA responses in *Arabidopsis thaliana*.
- Constitutive JA signaling in a *jaz* quintuple mutant (*jazQ*) was largely eliminated by mutations that block JA synthesis or perception. Comparison of *jazQ* and a *jazQ myc2 myc3 myc4* octuple mutant validated known functions of MYC2/3/4 in root growth, chlorophyll degradation, and susceptibility to the pathogen *Pseudomonas syringae*. We found that MYC TFs also control both the enhanced resistance of *jazQ* leaves to insect herbivory and restricted leaf growth of *jazQ*. Epistatic transcriptional profiles mirrored these phenotypes and further showed that triterpenoid biosynthetic and glucosinolate catabolic genes are up‐regulated in *jazQ* independently of MYC TFs.
- Our study highlights the utility of genetic epistasis to unravel the complexities of JAZ–TF interactions and demonstrates that MYC TFs exert master control over a JAZ‐repressible transcriptional hierarchy that governs growth–defense balance.

## Abstract 2

See also the Commentary on this article by Wasternack, **215**: 1291–1294.

## Introduction

Plants continuously integrate information from the environment to tailor their growth, development and defensive capabilities in ways that optimize fitness. Much of this phenotypic plasticity is orchestrated by the concerted action of a small number of plant hormones (Pieterse *et al*., 2009; Santner *et al*., 2009). Among the hormones whose biosynthesis and action is exquisitely tuned by changing environmental conditions is the oxylipin jasmonate (JA) (Browse, 2009; Bhosale *et al*., 2013). JA controls a multitude of transcriptional programs affecting plant growth, development, and responses to biotic and abiotic stress (Howe & Jander, 2008; Baldwin & Wu, 2010; Wasternack & Hause, 2013). In the past decade, tremendous progress has been made in understanding how JA regulates gene expression and also how JA responses are integrated with other signaling pathways (Pauwels & Goossens, 2011; Kazan & Manners, 2012; Campos *et al*., 2014; Huot *et al*., 2014; Chini *et al*., 2016).

When endogenous JA levels are below a threshold concentration, the expression of JA‐responsive genes is switched off through active repression of bHLH‐type MYC transcription factors (TFs) (Chini *et al*., 2016). This repression is mediated by JASMONATE ZIM‐DOMAIN (JAZ) proteins, which bind directly to MYCs to impede transcription by two distinct mechanisms (Chini *et al*., 2007; Thines *et al*., 2007; Yan *et al*., 2007). First, MYC‐bound JAZs recruit the corepressor TOPLESS (TPL) either directly (Shyu *et al*., 2012) or indirectly through the NOVEL INTERACTOR OF JAZ (NINJA) adaptor protein (Pauwels *et al*., 2010). Second, binding of the Jas motif of JAZ to the N terminus of MYC restricts access of MYC to the MED25 coactivator subunit of the mediator complex (Çevik *et al*., 2012; Chen *et al*., 2012; Zhang *et al*., 2015). A subset of JAZ repressors, including alternative splice variants of JAZ10, contain a cryptic MYC‐interaction domain (CMID) that tightly binds MYC and represses target gene expression (Chung & Howe, 2009; Chung *et al*., 2010; Moreno *et al*., 2013; Goossens *et al*., 2015; Zhang *et al*., 2017). Transcription of JA‐responsive genes is activated upon accumulation of jasmonoyl‐l‐isoleucine (JA‐Ile), the production of which is tightly controlled by environmental and developmental cues (Staswick & Tiryaki, 2004; Suza & Staswick, 2008; Koo & Howe, 2009). JA‐Ile promotes the formation of a nuclear co‐receptor complex consisting of the CORONATINE INSENSITIVE1 (COI1) F‐box protein and JAZ (Xie *et al*., 1998; Thines *et al*., 2007; Katsir *et al*., 2008; Melotto *et al*., 2008; Fonseca *et al*., 2009; Koo *et al*., 2009; Yan *et al*., 2009; Sheard *et al*., 2010). JA‐Ile‐dependent recruitment of JAZs to the E3 ubiquitin ligase Skp1‐Cullin‐F‐box protein (SCF)^COI1^ results in proteolytic destruction of JAZ repressors by the ubiquitin‐proteasome system, resulting in relief of transcriptional repression on MYC TFs (Chini *et al*., 2007; Thines *et al*., 2007).

A major gap in our understanding of JA signaling is how receptor activation maintains spatial and temporal control over diverse transcriptional outputs. Several lines of evidence suggest that the size and complexity of the JAZ–TF interactome may account, at least in part, for the diversity of JA responses. First, higher plants produce a large repertoire of JAZ proteins encoded by a family of *JAZ* genes (e.g. 13 in Arabidopsis), many of which are alternatively spliced to produce multiple JAZ isoforms (Vanholme *et al*., 2007; Yan *et al*., 2007; Chung & Howe, 2009; Chung *et al*., 2010; Bai *et al*., 2011; Thireault *et al*., 2015). Second, JAZs repress the transcriptional activity of not only MYCs (bHLH superfamily clade IIIe) but also several other bHLH TFs, as well as transcriptional regulators belonging to other TF families (Wager & Browse, 2012; Chini *et al*., 2016; Goossens *et al*., 2016). The combinatorial complexity resulting from interaction of multiple JAZs with multiple TFs could, in theory, explain much of the specificity and diversity of JA responses. Most JAZ–TF interactions, however, have been studied with *in vitro* approaches that alone are insufficient to delineate the biological consequences of specific JAZ–TF interactions. Among the factors that are likely to influence the biological outputs of JAZ–TF interactions are cell‐ and tissue type‐specific expression pattern, binding affinity of JAZ for TF targets, posttranslational modification, and recruitment of additional regulatory proteins to JAZ–TF complexes. It is therefore necessary to develop experimental approaches that provide insight into the *in vivo* function of specific JAZ–TF modules. Recent progress in this direction has come from studies showing that JAZ2 regulates a specific MYC‐dependent transcriptional cascade to modulate stomatal dynamics during pathogen infection (Gimenez‐Ibanez *et al*., 2017).

Here, we employed a genetic approach to define epistatic interactions between components of the core JA pathway and also to assess the contribution of the JAZ‐MYC signaling module to JA responses in Arabidopsis. Our experimental approach leveraged a *jaz* quintuple mutant (*jazQ*) that exhibits both enhanced responsiveness to exogenous JA and constitutive growth–defense antagonism as a consequence of mutations in *JAZ1*/*3*/*4*/*9*/*10* (Campos *et al*., 2016). We show that phenotypes of *jazQ* are largely dependent on intact pathways for JA biosynthesis and perception. Detailed phenotypic comparison between *jazQ* and a *jazQ myc2/3/4* octuple mutant demonstrated a key role for MYC TFs in restricting leaf growth concomitant with activation of leaf defense pathways, and also revealed JAZ‐repressible processes that do not require MYC TFs. Our collective data provide a genetic framework to understand how specific JAZ–TF transcriptional modules control discrete branches of the JA response.

## Materials and Methods

### Plant material and growth conditions

*Arabidopsis thaliana* (L.) Heynh. ecotype Columbia‐0 (Col‐0) was the wild‐type (WT) genetic background for all experiments. Construction of the quintuple *jaz* mutant (*jazQ*) and the *jazQ* suppressor screen has been described previously (Campos *et al*., 2016). *suppressor of jaz quintuple10* (*sjq10*) and *sjq66* suppressor mutants were identified by visual screening for larger rosette size from soil‐grown M_2_ plants. Phenotype heritability was confirmed in the M_3_ generation. To generate *jazQ allene oxide synthase* (*aos*) and *jazQ coi1*,* jazQ* was crossed to *aos* (Park *et al*., 2002) or to *coi1‐1*, respectively. *glabrous1* mutations were removed from *aos* and *coi1‐1* lines by backcrossing to Col‐0 (Yoshida *et al*., 2009). The *myc2 myc3 myc4* triple mutant (*mycT*) was generated by combining the previously described mutants *jin1‐7*/*myc2‐1* (SALK_040500) (Boter *et al*., 2004), *myc3‐1* (GK‐445B11) (Fernandez‐Calvo *et al*., 2011), and *myc4‐1* (GK‐491E10) (Fernandez‐Calvo *et al*., 2011). The *myc5‐1* mutant (SALK_060048) (Figueroa & Browse, 2015; Qi *et al*., 2015a) was used for construction of the *myc2345* quadruple mutant. Pedigrees describing the details of construction of *jazQ mycT* octuple and *myc2345* quadruple mutants are provided in Supporting Information Fig. S1. We note that this breeding scheme was not designed specifically for the construction of *jazQ mycT* and *myc2345*. Rather, the pedigrees shown were part of a broad strategy to combine mutations affecting all signaling components of the core JA pathway and to identify, in subsequent segregating populations, specific mutant combinations. PCR‐based genotyping of mutants was performed using primer sets flanking T‐DNA insertion sites, with a third primer specific for the T‐DNA border (Table S1) (Campos *et al*., 2016). Seeds were stratified for 3–4 d at 4°C before germination. Plants were grown in environmentally controlled chambers with cool‐white fluorescent light for all experiments. Unless stated otherwise, conditions were 21–20°C with a 16 h : 8 h, day (100 μE m^−2^ s^−1^) : night photoperiod.

### Measurements of shoot and root growth

**Figure 1:**
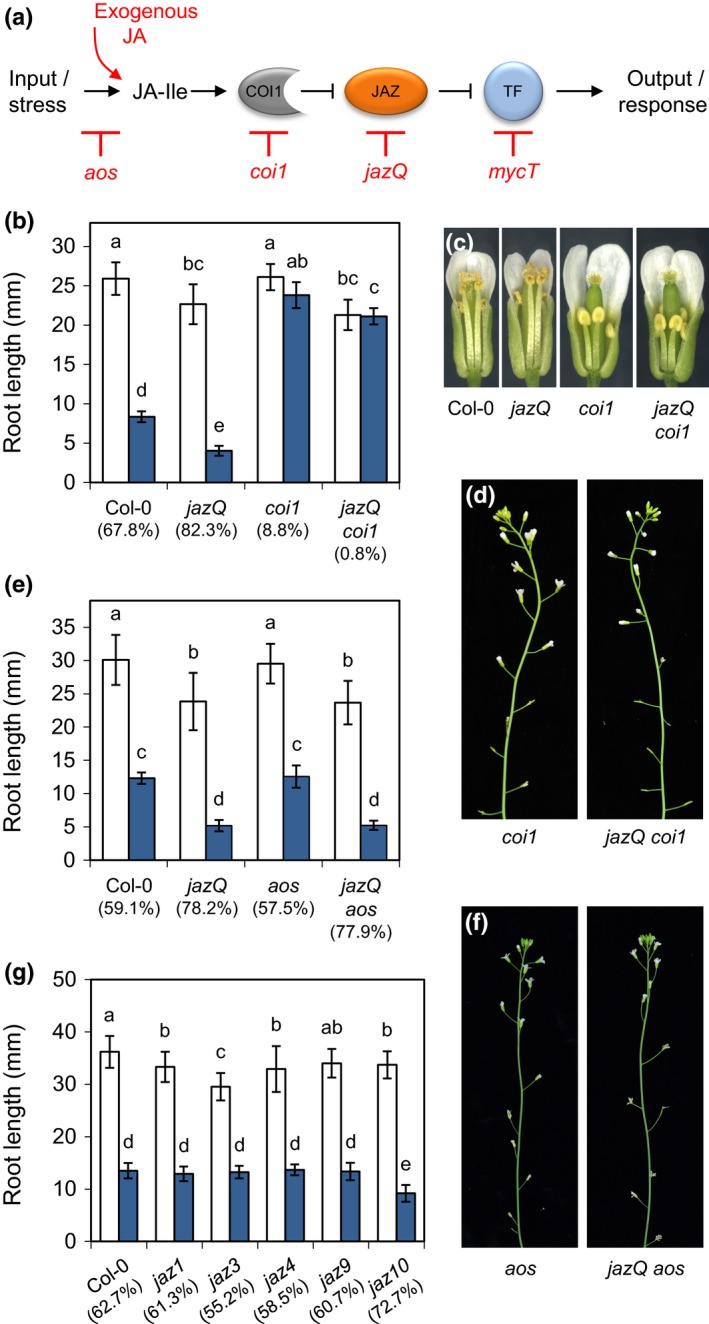
Genetic interaction between mutations affecting the core jasmonate (JA) response pathway. (a) Perturbations (shown in red) used in this study to manipulate JA responses included treatment with exogenous JA and loss‐of‐function mutations affecting JA biosynthesis (*allene oxide synthase* (*aos*)), the JA co‐receptor (*coronatine insensitive1* (*coi1*)), five JASMONATE ZIM‐DOMAIN (JAZ) repressors (*jazQ*), or three MYC transcription factors (*mycT*). (b, e, g) Root growth inhibition assays of Arabidopsis sextuple mutants of *jazQ* combined with (b) *coi1* or (e) *aos*, and of (g) *jaz1*,* jaz3*,* jaz4*,* jaz9* and *jaz10* single mutants. Root lengths were determined from seedlings grown on plates supplemented (closed bars) or not supplemented (open bars) with 25 μM methyl jasmonate (MeJA). Bars are means ± SD (*n *=* *7–24 seedlings per genotype). Per cent inhibition by MeJA is shown for each genotype in parentheses. Different letters represent significant differences at *P *<* *0.05 determined by two‐way ANOVA with Tukey's honest significant difference (HSD) test. Experiments were repeated twice with similar results. (c, d, f) Male sterility of Arabidopsis sextuple mutants of *jazQ* combined with (c, d) *coi1* or (f) *aos*. Anther filaments elongate and anthers dehisce in Col‐0 and *jazQ* flowers, but filaments do not elongate fully and anthers fail to dehisce in the sterile *coi1* and *jazQ coi1* flowers (c). Flowers of (d) *coi1* and *jazQ coi1* and of (f) *aos* and *jazQ aos* are sterile.

**Figure 2:**
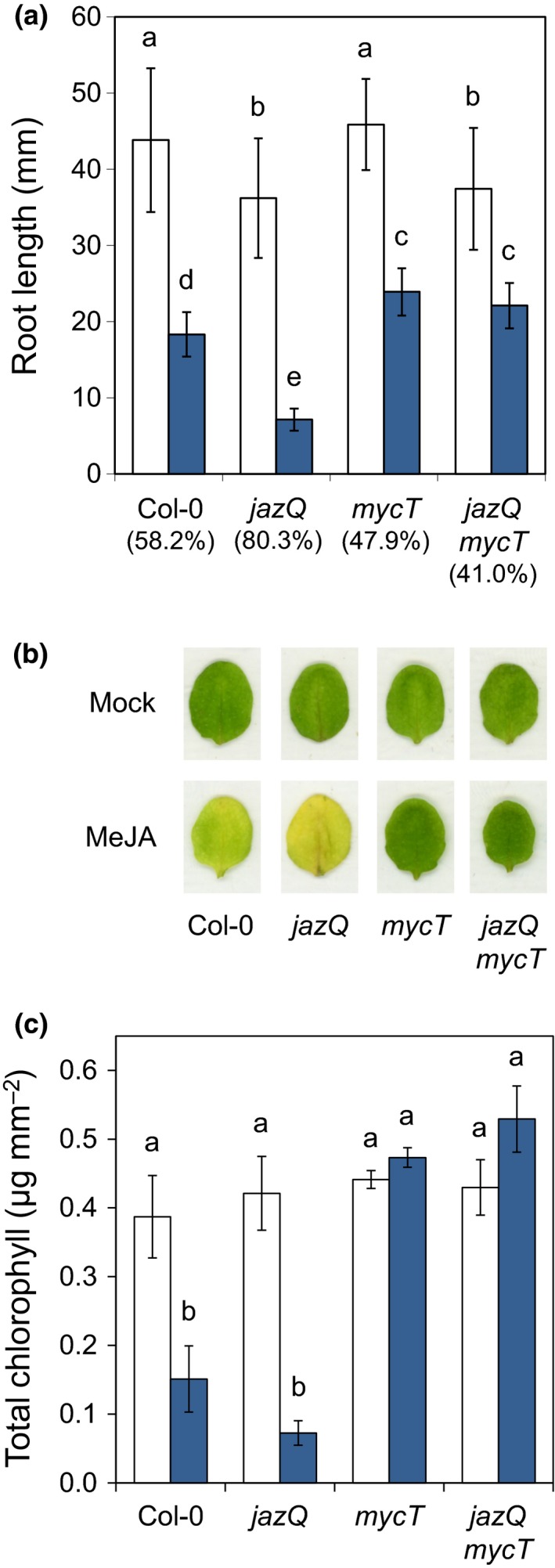
Jasmonate (JA) hypersensitivity of the *jasmonate zim‐domain* quintuple mutant (*jazQ*) depends on MYC2/3/4 transcription factors. (a) Root length of Arabidopsis seedlings grown on plates supplemented (closed bars) or not supplemented (open bars) with 25 μM methyl jasmonate (MeJA). Bars are means ± SD (*n *=* *33–48 seedlings per genotype). Per cent inhibition by MeJA is shown for each genotype in parentheses. (b) Photograph showing genotype‐dependent loss of chlorophyll in detached Arabidopsis leaves treated with 0 μM (mock) or 100 μM MeJA in the dark for 4 d. (c) Measurement of total chlorophyll levels in leaves treated as described in (b). Bars are means ± SD (*n *=* *3 leaves per genotype). Different letters represent significant differences at *P *<* *0.05 determined by two‐way ANOVA with Tukey's honest significant difference (HSD) test. Experiments were repeated three times with similar results.

Root growth inhibition assays (Shyu *et al*., 2012) were performed with seedlings grown on Petri plates (Thermo Fisher Scientific, Waltham, MA, USA) containing LS medium (0.5× Linsmaier and Skoog (Caisson Labs, Smithfield, UT, USA), 0.7% w/v phytoblend agar (Caisson Labs) and 0.8% w/v sucrose) supplemented with the concentration of methyl‐JA (MeJA; Sigma‐Aldrich) indicated in the legends to Figs 1 and 2. Primary root length of WT and mutant lines (grown on the same plate) was determined in 8‐ to 11‐d‐old seedlings using imagej software (http://imagej.nih.gov/ij/). Growth parameters, including leaf dry weight, leaf area, petiole length, rosette diameter, and flowering time, were determined as described previously (Campos *et al*., 2016).

### JA‐induced chlorophyll degradation assays

JA‐induced chlorophyll degradation assays were performed as previously described (Qi *et al*., 2015b). Briefly, third and fourth rosette leaves were gently removed from 3‐wk‐old plants and floated on distilled water or 100 μM MeJA, and were kept in the dark at 21°C for 4 d. For chlorophyll extraction, leaves were incubated overnight in methanol in the dark. Absorbance was measured at 652, 665 and 750 nm, and the chlorophyll content was calculated as previously described (Porra *et al*., 1989). Total chlorophyll was normalized to either leaf area or leaf fresh weight.

### Insect and pathogen assays

Insect feeding assays were performed as described previously (Herde *et al*., 2013; Campos *et al*., 2016). Plants were grown on soil at 20°C with an 8 h : 16 h, day (120 μE m^−2^ s^−1^) : night photoperiod. To each of 12 plants (6 wk old) per genotype, four neonate *Trichoplusia ni* larvae (Benzon Research, Carlisle, PA, USA) were reared for 10 d, after which larval weights were measured. *Pseudomonas syringae* (*Pst*) pv. DC3000 infection assays were performed as described previously (Katagiri *et al*., 2002). Plants were grown on soil at 22°C with a 12 h : 12 h, light (120 μE m^−2^ s^−1^) : dark photoperiod. Five‐week‐old Arabidopsis plants were dip‐inoculated with a *Pst* DC3000 suspension (1 × 10^8^ colony forming units (CFUs) ml^−1^) containing 0.025% Silwet L77 (Lehle Seeds, Round Rock, TX, USA). Bacterial population was determined by serial dilution and plating 4 d after inoculation.

### mRNA‐sequencing (RNA‐seq) analysis

Global gene expression profiling in 8‐d‐old whole seedlings (Col‐0 WT, *jazQ*,* mycT* and *jazQ mycT*) was assessed by mRNA sequencing (RNA‐seq), as described previously (Campos *et al*., 2016). *mycT* and *jazQ mycT* seedlings were grown and processed, and RNA‐seq analysis was performed in parallel with our previous analysis (Campos *et al*., 2016) of Col‐0, *jazQ*,* phytochrome B* (*phyB*), and *jazQ phyB* to facilitate cross‐comparisons; data for Col‐0 and *jazQ* are from Campos *et al*. (2016), while data for *mycT* and *jazQ mycT* are new here. Seedlings were grown in continuous light on solid medium supplemented with sucrose, and each sample was pooled from *c*. 200 seedlings, with three independent RNA samples (biological replicates) sequenced per genotype. Single‐end (50‐bp) sequencing was performed on the Illumina (San Diego, CA, USA) HiSeq 2000 platform at the Michigan State University Research Technologies Service Facility (https://rtsf.natsci.msu.edu). Filtered reads (Illumina quality control tools and Fastx toolkit; http://hannonlab.cshl.edu/fastx_toolkit/) were mapped to the arabidopsis information resource, genome release 10 (TAIR10) gene models with Rsem (v.1.2.11; default parameters; Li & Dewey, 2011). deseq (v.1.18.0; Anders & Huber, 2010) was used to normalize expected counts from Rsem and to assess differential gene expression relative to WT. The average transcripts per million (TPM) ± error and *P*‐values for all Arabidopsis genes are provided in Table S2. Gene onthology (GO) analysis of enriched functional categories was performed using David (v.6.7; Huang *et al*., 2009). The hypergeometric test with Benjamini & Hochberg's false discovery rate (FDR) correction was used to calculate over‐ and underrepresented GO categories among differentially expressed genes, using a *P*‐value < 0.05. RNA‐seq data are deposited at the National Center for Biotechnology Information Gene Expression Omnibus (GEO) as series record GSE98389.

### Photosynthesis measurements

Plants grown in plastic containers (‘Cone‐tainers’; Steuwe & Sons, Tangent, OR, USA) with an 8 h 19°C : 16 h 16°C, light (120 μE m^−2^ s^−1^) : dark period were used for gas exchange measurements (Campos *et al*., 2016). CO_2_ and light response curves were obtained from single mature rosette leaves (attached) of 8‐ to 10‐wk‐old plants on an LI‐6400XT system (Li‐Cor Biosciences, Lincoln, NE, USA) outfitted with a standard leaf chamber (chamber area = 6 cm^2^). Assimilation rates were normalized to projected leaf area as measured by image analysis with the Gimp software (www.gimp.org).

## Results

### Growth–defense antagonism in *jazQ* depends on jasmonate biosynthesis and perception

Allocation of metabolic resources to the production of plant defense compounds is often associated with reduced growth and biomass accumulation. These apparent growth–defense tradeoffs are of considerable interest for understanding plant form and function in both natural and agricultural ecosystems (Havko *et al*., 2016; Karasov *et al*., 2017; Züst & Agrawal, 2017). In our previous studies, we employed a JAZ‐depleted quintuple mutant (*jazQ*) to better understand how JA signaling balances growth and defense, and also to identify suppressor mutations that mitigate the antagonistic relationship between leaf growth and anti‐insect defense (Campos *et al*., 2016). This work showed that *phyB* mutations resulting in loss of the photoreceptor (phyB) rescue the reduced growth of *jazQ* rosette leaves but do not compromise the enhanced leaf defense traits of *jazQ*. Thus, the apparent tradeoff in *jazQ* between leaf biomass and defense can be effectively uncoupled through loss of phyB activity. The *jazQ* suppressor screen also identified a distinct class of mutants in which recovery of leaf growth was associated with visible loss of anthocyanin pigment in leaves. Two such *sjq* lines, *sjq10* and *sjq66*, exhibited features of male sterility that are associated with impaired JA biosynthesis or signaling (Fig. S2) (Thines *et al*., 2013). DNA sequencing of candidate genes in the JA pathway identified a C→T non‐sense mutation in codon 56 of the *AOS* gene in *sjq10* and a C→T missense mutation in codon 86 of the *COI1* gene in *sjq66* (Fig. S2). These results suggested that constitutive growth–defense antagonism (i.e. reduced growth and enhanced defense of shoots) resulting from the loss of JAZ1/3/4/9/10 in *jazQ* depends on intact pathways for JA biosynthesis and signaling.

To further investigate the relationship between *jazQ* and other components of the JA pathway (Fig. 1a), we reconstructed sextuple mutant lines in which *jazQ* was combined with mutant alleles of *aos* or *coi1*. Root growth inhibition assays showed that *jazQ coi1* plants resemble *coi1* single mutants in being strongly insensitive to JA treatment (Fig. 1b). We also found that *coi1* is epistatic to *jazQ* with respect to male sterility. Whereas *jazQ* flowers developed normally, *jazQ coi1* flowers had short anther filaments and lacked viable seed production (Fig. 1c,d). *jazQ coi1* flowers were also indehiscent at the time when stigmatic papillae are receptive to pollen for fertilization. Reconstructed *jazQ aos* lines maintained the JA hypersensitivity of *jazQ* plants and were also male sterile (Fig. 1e,f). These findings demonstrate that *coi1* and *aos* are epistatic to *jazQ* with respect to leaf growth and fertility traits, and that *coi1* abolishes the responsiveness of *jazQ* roots to exogenous JA.

### Constitutive growth repression of *jazQ* roots is independent of JA signaling

*jazQ* roots are not only hypersensitive to exogenous JA but also are shorter than WT roots in the absence of JA (Fig. 1b; Campos *et al*., 2016). To determine whether this constitutive short‐root phenotype results from increased sensitivity to endogenous JA, we compared the root length of *jazQ* seedlings grown in the absence of applied JA to that of *jazQ coi1* and *jazQ aos* plants. In contrast to our expectation, neither *coi1* nor *aos* rescued the short root of *jazQ* (Fig. 1b,e). This result indicated that constitutive root shortening in *jazQ* does not depend on JA signaling, and raised the possibility that one or more of the JAZs (JAZ1/3/4/9/10) affected by *jazQ* positively regulate root growth when JA concentrations are low. Comparison of *jaz* single mutants grown side by side on medium lacking JA showed that *jaz3* roots were significantly shorter than those of other mutants (Fig. 1g), suggesting that JAZ3 promotes root growth under these conditions. Only *jaz10‐1* roots were shorter than WT roots on medium containing JA, as shown in previous studies (Demianski *et al*., 2012; Moreno *et al*., 2013).

### Enhanced responsiveness of *jazQ* to exogenous JA requires MYC2/3/4

Among the diverse TFs that physically interact with JAZs are MYC2 and the closely related proteins MYC3 and MYC4, which perform prominent roles in JA signaling (Dombrecht *et al*., 2007; Fernandez‐Calvo *et al*., 2011; Schweizer *et al*., 2013). MYC5, which is also a member of the group IIIe subfamily of bHLH TFs to which MYC2/3/4 belong, plays a role in JA‐mediated stamen development (Figueroa & Browse, 2015; Qi *et al*., 2015a). We assessed whether MYC5 might contribute to JA signaling in nonfloral tissues by comparing responses of vegetative organs of a *myc2 myc3 myc4* triple mutant (referred to hereafter as *mycT*) to those of a *myc2 myc3 myc4 myc5* quadruple mutant (*myc2345*). No differences in root growth, leaf growth, or leaf anthocyanin concentrations were observed between *mycT* and *myc2345* (Fig. S3). Subsequent studies were thus focused on investigating the genetic interaction between *jazQ* and *mycT*.

To investigate the collective role of MYC2/3/4 in *jazQ* phenotypes, we compared JA responses in *jazQ* to those in *mycT* and the corresponding *jazQ mycT* octuple mutant. Root growth assays performed on JA‐free medium showed that *mycT* and WT roots were similar in length, whereas *jazQ mycT* plants displayed the constitutive short‐root trait of *jazQ* (Fig. 2a). Assays performed on JA‐supplemented media showed that *mycT* roots were partially insensitive to JA (Fig. 2a), consistent with previous studies (Lorenzo *et al*., 2004; Fernandez‐Calvo *et al*., 2011; Gasperini *et al*., 2015). Moreover, the response of *jazQ mycT* roots to JA was indistinguishable from that of *mycT* plants. These data indicate that the hypersensitivity of *jazQ* roots to JA is mediated by MYC2/3/4 but that the constitutive short‐root phenotype of *jazQ* does not require these TFs.

We used a dark‐ and JA‐induced chlorophyll degradation assay to investigate how *jazQ* and *mycT* interact to mediate shoot responses to exogenous JA. Control experiments showed comparable levels of chlorophyll in WT, *jazQ*,* mycT* and *jazQ mycT* plants, in both intact rosette leaves and in detached leaves incubated in the dark in the absence of exogenous JA (Fig. S4; Table 1). Exogenous JA promoted chlorophyll degradation in detached WT leaves incubated in the dark (Fig. 2b,c), as previously reported (Zhu *et al*., 2015). JA‐induced leaf degreening was modestly but reproducibly exacerbated in *jazQ* leaves relative to Col‐0, and this effect was abolished in both *mycT* and *jazQ mycT* lines. These results show that the responsiveness of *jazQ* leaves to exogenous JA is dependent on MYC2/3/4.

**Table 1:** Chlorophyll and anthocyanin content in plants with *jasmonate zim‐domain* quintuple (*jazQ*) and *myc2 myc3 myc4* triple (*mycT*) mutations

	Col‐0	*jazQ*	*mycT*	*jazQ mycT*
Total chlorophyll^a^	1.37 ± 0.10	1.33 ± 0.09	1.34 ± 0.07	1.41 ± 0.15
A530/g FW^b^	0.97 ± 0.30	3.75† ± 0.80	0.67 ± 0.25	1.83† ± 0.47

a Total chlorophyll levels (μg chlorophyll per mg fresh tissue) quantified from rosette leaves of 21‐d old plants (*n* = 10). Plant genotype had no effect at *P* < 0.05 with an ANOVA. Experiment was repeated three times with similar results.

b Anthocyanin levels were quantified from rosette leaves of 21‐d old plants (*n* = 14). Different symbols denote significant differences at *P* < 0.05 with Tukey's HSD test. Experiment was repeated four times with similar results.

### Growth and defense responses in *jazQ* leaves are largely dependent on MYC TFs

**Figure 3:**
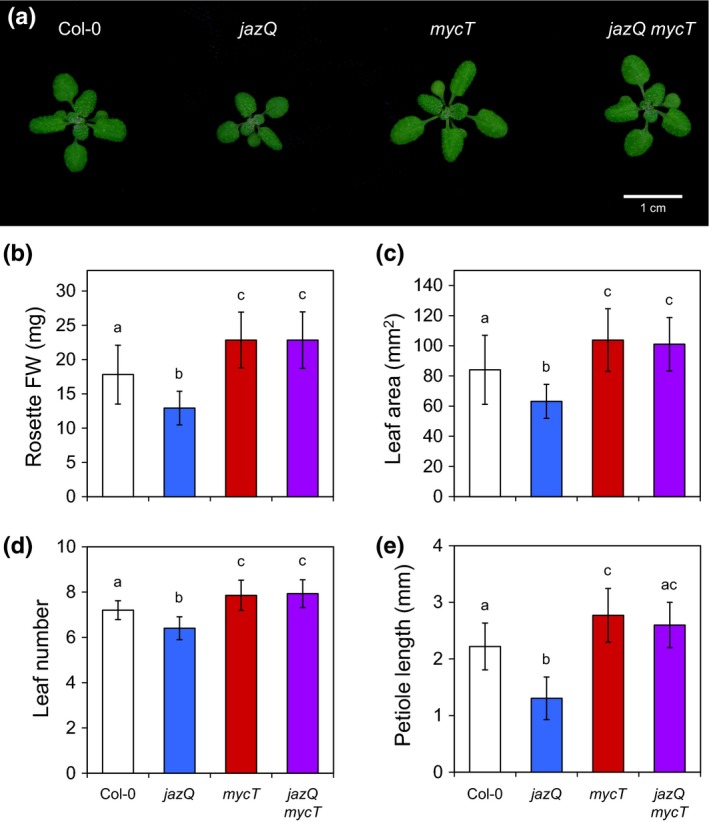
Growth suppression of *jasmonate zim‐domain* quintuple mutant (*jazQ*) rosette leaves is mediated by MYC transcription factors. (a) Photograph of Col‐0, *jazQ*,* mycT* and *jazQ mycT* rosettes of 21‐d‐old Arabidopsis plants. (b–e). Rosette growth at 21 d was assessed by measuring (b) biomass, (c) leaf area, (d) number of leaves, and (e) petiole length. Bars are means ± SD (*n *=* *14–15 plants per genotype). Different letters represent significant differences at *P *<* *0.05 determined by two‐way ANOVA with Tukey's honest significant difference (HSD) test. The experiment was repeated four times with similar results.

To determine whether MYC2/3/4 activity plays a role in JA‐mediated restriction of shoot growth, we compared leaf growth traits of 21‐d‐old plants grown in soil under our standard conditions. Consistent with previous studies (Campos *et al*., 2016), the shoot biomass, projected leaf area, petiole length, and leaf number were all decreased in *jazQ* relative to WT (Fig. 3). Loss of MYC2/3/4 in *mycT* plants had the opposite effect on shoot growth, such that leaf area, biomass and petiole length of *mycT* plants were greater than those of WT. Significantly, loss of MYCs in the *jazQ* genetic background completely recovered the restricted growth of *jazQ* leaves, and the area and biomass of *jazQ mycT* leaves also exceeded those of WT (Fig. 3b–e). These data demonstrate that JAZ1/3/4/9/10 and MYC2/3/4 act as positive and negative regulators, respectively, of leaf growth and biomass accumulation.

**Figure 4:**
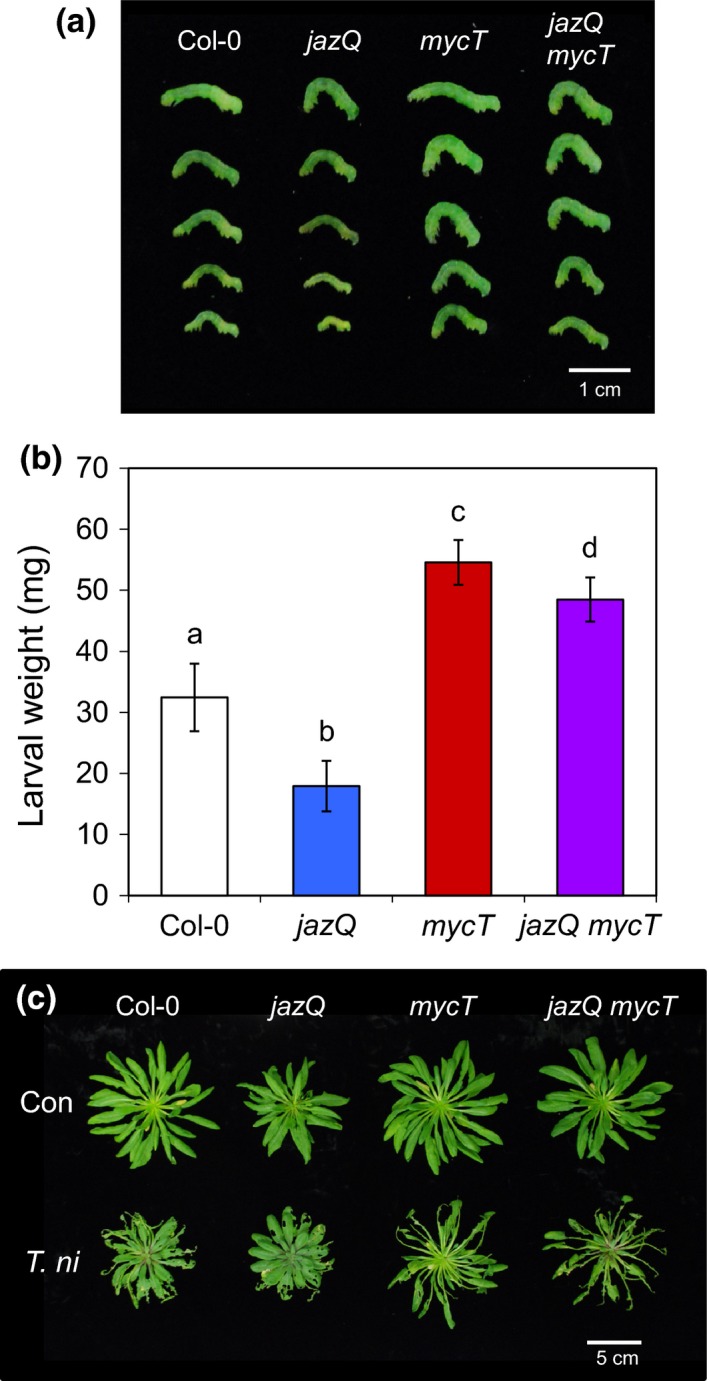
MYC2/3/4 are required for increased resistance of the *jasmonate zim‐domain* quintuple mutant (*jazQ*) to a lepidopteran herbivore. Arabidopsis plants of the indicated genotype were challenged with neonate *Trichoplusia ni* larvae. Larval weights were measured 10 d later. (a) Photograph of representative *T. ni* larvae at the end of the feeding trial. (b) Larval weight at the end of the feeding trial. Bars are means ± SD (*n *=* *12, where each sample is the mean of four larvae per plant). Different letters represent significant differences at *P *<* *0.05 with Tukey's honest significant difference (HSD) test. (c) Photograph of control (Con) and insect‐challenged plants at the end of the feeding trial. The experiment was repeated three times with similar results.

We next determined whether MYC TFs are required for enhanced resistance of *jazQ* plants to insect herbivory (Campos *et al*., 2016). Feeding assays performed with *Trichoplusia ni* (cabbage looper) showed that, as expected, larval growth on *jazQ* plants was reduced relative to growth of WT‐reared insects. By contrast, the mass of larvae reared on either *mycT* or *jazQ mycT* plants exceeded that on WT (Fig. 4a,b). These differences in caterpillar performance were generally reflected by the amount of leaf tissue consumed during the course of the bioassay (Fig. 4c). We conclude that enhanced defense of *jazQ* to *T. ni* feeding is dependent on MYC2/3/4 TFs. The finding that the weight gain of caterpillars grown on *mycT* slightly exceeded that of larvae reared on *jazQ mycT* plants (Fig. 4b) suggests that other regulatory factors may contribute to *jazQ*‐mediated anti‐insect defense. We also tested the effect of *mycT* on leaf anthocyanin concentrations, which are elevated in *jazQ* (Campos *et al*., 2016). Anthocyanin concentrations in *mycT* leaves were modestly but consistently lower than those in WT. Leaf anthocyanin concentrations in *jazQ mycT* were intermediate between those of *jazQ* and WT (Table 1). These results indicate that, although MYC2/3/4 positively regulate anthocyanin production, these TFs are not sufficient to account for all anthocyanin accumulation in *jazQ*.

**Figure 5:**
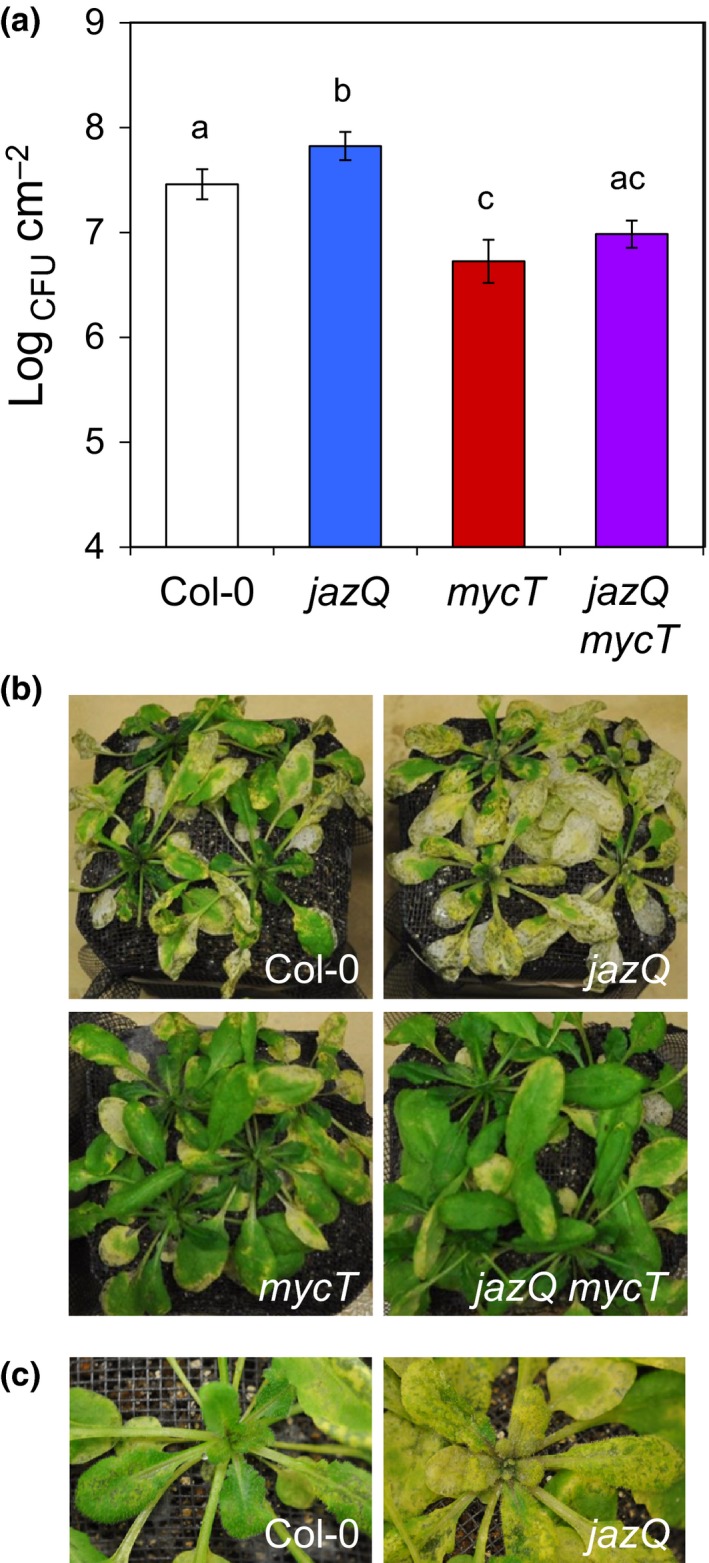
Enhanced susceptibility of the *jasmonate zim‐domain* quintuple mutant (*jazQ*) to bacterial infection requires MYC2/3/4. Five‐week‐old Arabidopsis plants of the indicated genotype were dip‐inoculated with *Pseudomonas syringae* pv. DC3000 (*Pst* DC3000) at 1 × 10^8^ colony forming units (CFUs) ml^−1^. (a) Bacterial populations, represented as CFUs, in fully expanded leaves were determined 3 d after inoculation. Data show the mean ± SD (*n *=* *4 technical replicates). Different letters represent significant differences at *P *<* *0.05 with Tukey's honest significant difference (HSD) test. The experiment was repeated three times with similar results. (b) Photograph of plants taken 6 d after inoculation with *Pst* DC3000. (c) Zoom‐in images to show increased symptom development on young leaves of *jazQ* plants 4 d after inoculation.

The bacterial pathogen *P. syringae* pv. tomato DC3000 (*Pst* DC3000) exploits the host JA signaling pathway to promote virulence. We therefore sought to determine whether the constitutive activation of JA signaling by *jazQ* is sufficient to alter host susceptibility to this pathogen, which uses the JA‐Ile mimic coronatine as part of its virulence strategy to suppress host defenses (Melotto *et al*., 2006). Indeed, bacterial infection assays showed that *Pst* DC3000 multiplies to a higher level on *jazQ* than on WT leaves (Fig. 5a,b). The enhanced susceptibility of *jazQ* was particularly evident from strong disease symptoms in young emerging leaves, which in WT plants typically show few disease symptoms after *Pst* DC3000 infection (Fig. 5c). Consistent with previous reports (Laurie‐Berry *et al*., 2006; Fernandez‐Calvo *et al*., 2011), *mycT* plants were more resistant than WT to *Pst* DC3000 infection. Moreover, we found that *jazQ mycT* plants resisted *Pst* DC3000 infection to a similar level to *mycT* (Fig. 5a). These results show that MYCs TFs are required for the increased susceptibility of *jazQ* to *Pst* DC3000 infection, and are consistent with the idea that this pathogen activates the JA pathway as part of a virulence strategy to suppress salicylate‐based immunity (Zhao *et al*., 2003; Katsir *et al*., 2008; Fernandez‐Calvo *et al*., 2011; Demianski *et al*., 2012; Zheng *et al*., 2012).

### MYC TFs do not promote delayed flowering in *jazQ*

**Figure 6:**
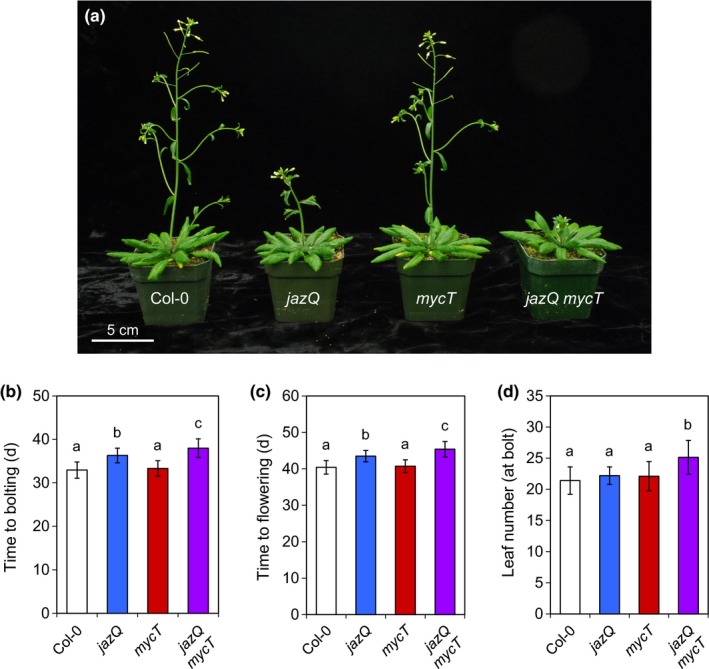
Delayed flowering of the *jasmonate zim‐domain* quintuple mutant (*jazQ*) is not dependent on MYC transcription factors. (a) Photograph of Col‐0, *jazQ*,* mycT* and *jazQ mycT* inflorescence in 45‐d‐old Arabidopsis plants. (b, c) Flowering time was assessed by counting the days required for (b) bolting and (c) flowering. (d) Quantification of the number of leaves at the time of bolting. Bars are means ± SD (*n *=* *29–32 plants per genotype). Different letters represent significant differences at *P *<* *0.05 with Tukey's honest significant difference (HSD) test. The experiment was repeated four times with similar results.

Several studies have reported that JA signaling through the COI1‐JAZ pathway delays the onset of flowering in Arabidopsis (Robson *et al*., 2010; Yang *et al*., 2012; Song *et al*., 2013; Zhai *et al*., 2015). Consistent with these observations, we found that *jazQ* plants are developmentally delayed in flowering but have the same number of leaves as WT at the time of bolting (Fig. 6; Campos *et al*., 2016). Under our standard long‐day growth conditions, *mycT* alone did not have an obvious effect on flowering time. Unexpectedly, however, the combination of *jazQ* and *mycT* retarded flowering time even later than *jazQ*, and also increased the number of leaves at the time of bolting (Fig. 6). These data indicate that MYC2/3/4 do not mediate the delayed flowering of *jazQ*.

### Global transcript profiling identifies MYC‐dependent and ‐independent sectors of JAZ‐repressible gene expression

**Figure 7:**
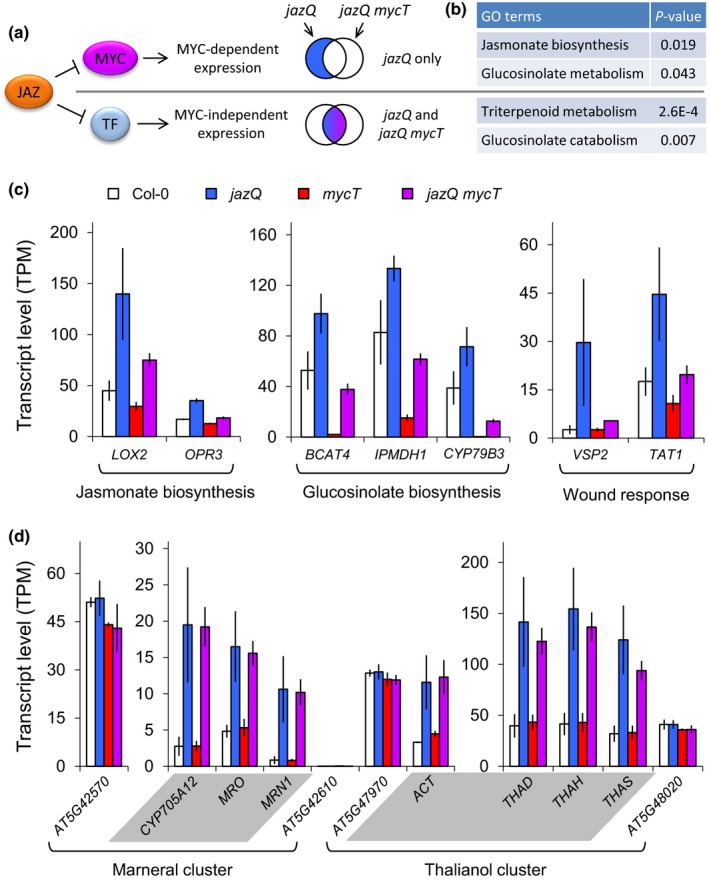
JASMONATE ZIM‐DOMAIN (JAZ) proteins coordinate gene expression through MYC‐dependent and ‐independent mechanisms in Arabidopsis. (a) Conceptual framework of genes up‐regulated in *jazQ*. Genes controlled by the JAZ‐MYC module are up‐regulated in *jazQ* but not *jazQ mycT*, whereas genes controlled by other (MYC‐independent) JAZ‐transcription factor (TF) modules are up‐regulated in both *jazQ* and *jazQ mycT*. (b) Gene ontology (GO) terms of genes up‐regulated in *jazQ* only (top) or in *jazQ* and *jazQ mycT* (bottom). (c) MYC‐dependent expression of selected genes associated with jasmonate biosynthesis, glucosinolate biosynthesis, and the wound response. *lipoxygenase 2* (*LOX2*), *branched‐chain aminotransferase 4* (*BCAT4*), *isopropyl malate dehydrogenase 1* (*IPMD1*), *CYP79B3* and *TAT1* promoters are reported targets of MYC2. (d) MYC‐independent expression of genes associated with triterpenoid metabolism from the marneral and thalianol clusters (shaded genes), with genes flanking these clusters shown for comparison. Errors bars are ± SD of the mean of three biological replicates. Expression levels are transcripts per million (TPM).

To better understand the contribution of MYC TFs to changes in gene expression resulting from loss of JAZ1/3/4/9/10, we used mRNA sequencing (RNA‐seq) to compare transcript profiles of WT, *jazQ*,* mycT*, and *jazQ mycT* seedlings grown in the absence of exogenous JA (Table S2). We used stringent statistical criteria to define a set 99 JAZ1/3/4/9/10‐repressible, MYC2/3/4‐inducible transcripts whose abundance relative to Col‐0 is higher in *jazQ* but not in *jazQ mycT* (Figs 7a, S5). Likewise, 159 MYC‐independent genes were identified as being up‐regulated in both *jazQ* and *jazQ mycT* (Figs 7a, S5). Based on this analysis, we estimate that MYC2/3/4 activity is required for the increased expression of *c*. 38% of all genes that are up‐regulated in *jazQ* seedlings. Among the 258 genes that were up‐regulated in *jazQ* relative to Col‐0, the MYC‐dependent gene set was associated with gene ontologies for JA biosynthesis and glucosinolate (GLS) metabolism, and also included known wound‐response genes such as *vegetative storage protein 2* (*VSP2*) and *tyrosine aminotransferase 1* (*TAT1*) (Fig. 7b,c). That several of these MYC‐dependent genes, most notably genes involved in GLS biosynthesis, were strongly repressed by *mycT* relative to Col‐0 (Figs 7c, S6) is consistent with a role for MYC TFs in maintaining basal expression of JA‐responsive genes.

Among the genes that were up‐regulated most robustly in *jazQ* (relative to WT) and independently of MYC2/3/4 were those associated with triterpenoid biosynthesis and GLS catabolism (Fig. 7b). Marneral and thalianol triterpenoids are synthesized in the Arabidopsis root epidermis by enzymes encoded within metabolic gene clusters (Field & Osbourn, 2008). Strikingly, transcript levels for all genes within these clusters were similarly elevated in both *jazQ* and *jazQ mycT*, and *mycT* alone did not affect the basal expression level observed in WT (Fig. 7d). We also found that the expression of genes immediately flanking the marneral and thalianol gene clusters was not altered by *jazQ* or *mycT*. Thus, the effect of *jazQ* in increasing the expression of triterpenoid biosynthetic genes is not only independent of MYC2/3/4, but is also spatially restricted to genes within the clusters.

In intact plant cells, GLSs are stored as inert glycosides physically separated from GLS catabolic enzymes (Halkier & Gershenzon, 2006). Tissue disruption by herbivores allows myrosinases to deglycosylate GLSs, whereas ancillary specifier proteins catalyze the formation of cyanate and nitrile toxins from the corresponding aglycones (Fig. S7a). We found that genes encoding enzymes involved in GLS breakdown were significantly up‐regulated in both *jazQ* and *jazQ mycT* (Fig. S7b,c). This expression pattern was highly distinct from that of MYC‐dependent GLS biosynthetic genes (Figs S6, S7), suggesting that specific JAZ–TF modules control different aspects of GLS metabolism. Further analysis of the broader set of 159 genes that are up‐regulated in *jazQ* independently of MYC2/3/4 (Fig. 7a) revealed a substantial overlap with genes that are up‐regulated in roots of the *ninja‐1* mutant (Fig. S5c) (Gasperini *et al*., 2015). This overlap was significantly greater than the portion of MYC‐dependent genes that overlapped with *ninja‐1* (*P *=* *0 .0013; Fisher's exact test), suggesting that NINJA cooperates with JAZ1/3/4/9/10 to repress the expression MYC‐independent genes.

Among the 88 genes expressed to higher levels in *mycT* than Col‐0, there was little overlap with genes misregulated by either *jazQ* or *jazQ mycT* (Fig. S5a). We found that the majority of genes (37/72 genes; 51.4%) uniquely up‐regulated in *mycT* are associated with chloroplast processes and, in particular, photosynthesis (Fig. S8). Gas exchange experiments showed that *mycT* leaves had higher CO_2_ assimilation rate per unit leaf area than Col‐0 leaves under our plant growth conditions (Fig. S9). Photosynthetic rates measured in *jazQ* and *jazQ mycT* leaves were comparable to those in WT (Fig. S9). These findings suggest a potential role for the MYC2/3/4 TFs as negative regulators of photosynthesis, and further highlight the complex relationship between JA signaling and photosynthetic capacity (Campos *et al*., 2016).

## Discussion

### Genetic interactions in the core JA signaling pathway

Genetic epistasis provides a powerful approach to dissect complex interactions between core components of the JA pathway, as exemplified by recent studies of root responses to JA (Acosta *et al*., 2013; Gasperini *et al*., 2015). We previously employed a genetic suppressor screen to identify mutations that uncouple growth–defense antagonism in leaves of the *jazQ* mutant (Campos *et al*., 2016). Here, we report on a new class of *jazQ* suppressor mutants in which defects in either JA‐Ile biosynthesis (*aos*) or perception (*coi1*) suppress both the slow growth and enhanced defense traits of *jazQ* leaves. Given that JA‐Ile biosynthesis and perception are both required for turnover of JAZ repressors, the most likely mechanistic explanation for the observed *jazQ aos* and *jazQ coi1* phenotypes is that one or more of the remaining JAZs in *jazQ* are stabilized by the loss of the JAZ degradation machinery. This model implies that the remaining complement of JAZs in *jazQ* can strongly repress JA responses in the absence of JAZ1/3/4/9/10, thus providing evidence for functional redundancy among JAZs. The genetic interactions defined in our study are generally consistent with current models of JA‐mediated signal transduction in which JA‐Ile and COI1 work together to degrade JAZs, thereby relieving repression on target TFs (Fig. 1a).

A unique attribute of *jazQ* in comparison to *jaz* single mutants is its enhanced sensitivity to exogenous JA. One explanation for this phenotype is that genetic depletion of JAZs increases the capacity of SCF^COI1^ to ubiquitylate the remaining pool of JAZ substrates in *jazQ*, which is consistent with evidence that COI1 dosage modulates sensitivity to JA (Feng *et al*., 2003). Alternatively, the enhanced responsiveness of *jazQ* to JA treatment may reflect the loss of JAZ proteins that desensitize JA responses once the signaling pathway is initiated (Campos *et al*., 2014). JAZ1 and JAZ10 probably contribute to this role. First, JAZ10 alternative splice variants are resistant to JA‐induced degradation, and loss of these isoforms is associated with increased sensitivity to JA (Chung & Howe, 2009; Moreno *et al*., 2013). Second, the N termini of both JAZ10 and JAZ1 contain a cryptic MYC‐interaction domain (CMID) that may enhance the capacity of these proteins to repress MYCs in JA‐elicited cells (Moreno *et al*., 2013; Goossens *et al*., 2015; Zhang *et al*., 2017). The up‐regulation of JA biosynthetic genes in *jazQ* raises the additional possibility that changes in endogenous JA/JA‐Ile concentrations contribute to the phenotypes observed in this line. Regardless of the mechanisms that confer enhanced sensitivity of *jazQ* to JA, it is evident that *jazQ* mutant phenotypes are relatively subtle in comparison to WT plants subject to chronic JA exposure. This observation supports the notion of genetic redundancy among *JAZ* genes and provides a strong rationale for further analysis of higher order *jaz* mutants.

In addition to JA hypersensitivity, *jazQ* roots are *c*. 25% shorter than those of WT in the absence of exogenous JA (Campos *et al*., 2016; this work). The unexpected finding that constitutive root shortening also occurs in *jazQ aos*,* jazQ coi1*, and *jazQ mycT* mutants indicates that this phenotype is not dependent on core components of the JA pathway. Analysis of *jaz* single mutants further suggested that JAZ3 may serve a role in promoting root growth in the absence of JA, but additional studies are needed to test this hypothesis. We are not aware of other studies suggesting JA‐independent roles for JAZ proteins. However, it was recently reported that overexpression of Arabidopsis TIFY8, a ZIM domain‐containing protein belonging to the TIFY family to which JAZs belong, stunts primary root growth in the absence of exogenous JA (Perez *et al*., 2014). TIFY8 also represses transcriptional activity through recruitment of the NINJA−TPL corepressor complex, but does not affect the expression of JA‐responsive genes. Acosta *et al*. (2013) found that null mutations in *NINJA* led to JA‐independent reduction in root cell elongation to generate a short‐root phenotype similar to that of *jazQ*. Given that JAZs and NINJA physically interact and negatively regulate JA signaling in roots (Pauwels *et al*., 2010; Acosta *et al*., 2013), it is possible that JAZ−NINJA complexes have a role in promoting root growth under specific conditions or in specific cell types. Unlike *jazQ*,* ninja* null mutations do not confer hypersensitivity to exogenous JA (Acosta *et al*., 2013), presumably because loss of NINJA does not impede JA‐Ile‐dependent JAZ degradation via SCF^COI1^ and the 26S proteasome. In future studies it will be informative to determine whether *jazQ* and *ninja* mutations mediate constitutive root shortening through the same or parallel pathways.

### Unraveling physiological roles of multiple JAZ–TF interactions

**Figure 8:**
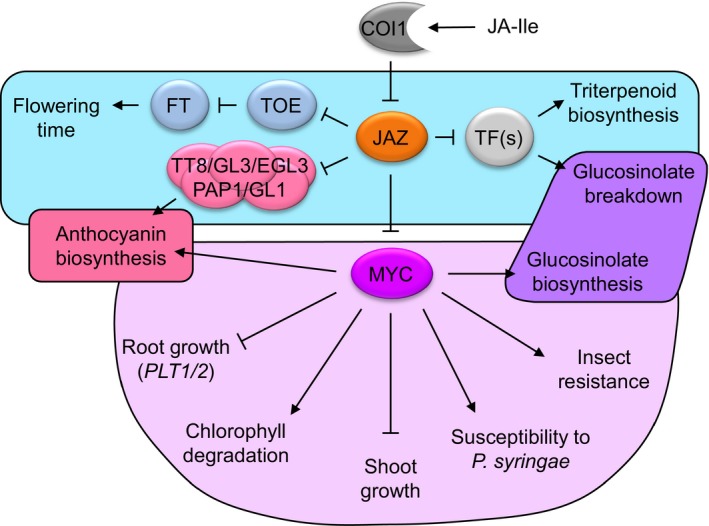
Conceptual model of how jasmonate (JA) signaling controls multiple JASMONATE ZIM‐DOMAIN (JAZ)‐transcription factor modules to mediate diverse physiological responses. The model depicts specific processes that are either dependent (light purple) or not dependent (blue) on MYC transcription factors (TFs). Some processes, including glucosinolate and anthocyanin metabolism, are controlled by JAZ‐mediated repression of both MYC and non‐MYC TFs. See the Discussion section for details. COI1, coronatine insensitive 1; FT, flowering locus T; TOE, target of eat; TT8, transparent testa 8; GL3, glabrous 3; GL1, glabrous 1; PAP1, production of anthocyanin pigment 1; EGL3, enhancer of glabra 3; TF, transcription factor; PLT1/2, plethora 1 and 2.

A current challenge in JA research is to understand how the hormone controls diverse aspects of plant growth, development, and responses to the environment. As summarized in Fig. 8, our genetic data suggest that JAZ‐mediated control over MYC2/3/4 TFs plays a major role in executing the majority of JA‐related phenotypes in *jazQ*. We found that MYCs are largely responsible for root hypersensitivity to JA, reduced leaf biomass, leaf defense against insect herbivory, and JA‐induced chlorophyll degradation. The latter observation supports previous studies showing that exogenous JA and leaf damage reduce the abundance of photosynthetic proteins in Arabidopsis leaves, perhaps as a strategy to mobilize resources for defense (Gfeller *et al*., 2011; Shan *et al*., 2011; Zhu *et al*., 2015). Consistent with previous work showing that MYCs are critical for the effects of coronatine on virulence of *Pst* DC3000 (Fernandez‐Calvo *et al*., 2011; Zheng *et al*., 2012; Schweizer *et al*., 2013; Gimenez‐Ibanez *et al*., 2017), we also found that *jazQ*‐mediated enhanced susceptibility to this pathogen is eliminated by *mycT*. These collective data support a dominant role for MYC TFs in mediating JA responses in Arabidopsis and further demonstrate that MYC activity can be enhanced through loss of a specific subset of MYC‐interacting JAZs *in vivo*.

Our findings support an increasing number of studies showing that JAZs functionally interact with TFs other than MYCs (Fig. 8) (Wager & Browse, 2012; Chini *et al*., 2016; Goossens *et al*., 2016). One example is *jazQ*‐mediated anthocyanin accumulation, which was reduced but not eliminated by *mycT*. The role of MYC TFs as positive regulators of anthocyanin biosynthesis is well established (Niu *et al*., 2011; Nakata *et al*., 2013). Other JAZ‐interacting TFs, however, including YABBY, MYB, and transparent testa 8/glabrous 3, also contribute to JA‐inducible anthocyanin accumulation (Shan *et al*., 2009; Qi *et al*., 2011; Boter *et al*., 2015). It thus seems likely that these non‐MYC TFs contribute to the anthocyanin accumulation observed in *jazQ mycT* plants (Fig. 8).

We also demonstrate that MYC2/3/4 activity is not required for delayed flowering of *jazQ*. This finding agrees with recent models in which a subset of JAZ proteins (i.e. JAZ1/3/4/9) repress TARGET OF EAT1 (TOE1) and TOE2 TFs, which delay flowering time by repressing the expression of *FLOWERING LOCUS T* (*FT*) (Zhai *et al*., 2015). Although additional work is needed to understand how *mycT* further delays the flowering time of *jazQ*, inspection of RNA‐seq data (Campos *et al*., 2016; this work) showed a strong correlation between *FT* transcript levels and flowering time in various mutant lines. For example, *FT* mRNA levels were high in the early‐flowering *phyB* and *jazQ phyB* lines, whereas *FT* transcript levels were strongly reduced (relative to Col‐0) in *jazQ* and even lower in *jazQ mycT* (Fig. S10).

### Control of specialized metabolism by JAZ repressors

Although JA has long been recognized as a potent elicitor of specialized metabolism (Gundlach *et al*., 1992), the underlying transcriptional mechanisms that control these metabolic pathways in specific cell types and morphological structures are only beginning to be elucidated (De Geyter *et al*., 2012). Consistent with previous studies (Schweizer *et al*., 2013; Campos *et al*., 2016), our results establish a direct link between JAZ1/3/4/9/10 and their interacting MYC TFs in controlling GLS production. Interestingly, however, we also found that genes encoding myrosinases and associated specifier proteins involved in GLS breakdown are up‐regulated in *jazQ* independently of MYC2/3/4, suggesting that additional JAZ‐interacting TFs govern distinct aspects GLS metabolism (Fig. 8). This interpretation is consistent with recent work showing that GLS accumulation is dependent on a complex transcriptional network in which many TFs optimize metabolite accumulation across multiple tissue types and environmental conditions, thus allowing for tight yet flexible control of the ‘mustard oil bomb’ (Li *et al*., 2014).

Our results also provide new insight into processes underlying the expression of metabolic gene clusters for triterpenoid biosynthesis. We found that genes within the thalianol and marnerol clusters are coordinately up‐regulated in *jazQ* seedlings, which is consistent with the ability of JA to elicit triterpenoid production (Hayashi *et al*., 2003). MYC2‐like TFs are implicated in the control of metabolic gene clusters in several plant species (Shang *et al*., 2014; Cardenas *et al*., 2016). However, our finding that *mycT* does not suppress the elevated expression of the thalianol and marnerol clusters in *jazQ* argues against a role for MYC2/3/4 in controlling these genes. The observation that genes within these clusters are strongly up‐regulated in roots of *ninja* mutants (Gasperini *et al*., 2015) further supports the notion that recruitment of co‐repressors by JAZ‐NINJA complexes (Pauwels *et al*., 2010) may negatively regulate triterpenoid production. Indeed, recent studies show that chromatin modifications within the thalianol and marnerol clusters are associated with silencing and activation of individual cluster genes (Nützmann *et al*., 2016; Yu *et al*., 2016). Mutants affected in NINJA and JAZ function may provide useful tools to delineate the contribution of core JA signaling components to chromatin signatures and TF modules that coordinate the expression of metabolic gene clusters.

### MYC TFs mediate repression of leaf growth

JA is a potent inhibitor of leaf growth and biomass accumulation in Arabidopsis (Yan *et al*., 2007; Zhang & Turner, 2008; Noir *et al*., 2013; Attaran *et al*., 2014). Similar effects are observed in monocots (Yang *et al*., 2012; Hibara *et al*., 2016), suggesting that the underlying pathways for JA‐mediated restriction of leaf growth are conserved. JA acts primarily to reduce leaf cell number through perturbation of the cell cycle but effects on leaf cell expansion have also been documented (Zhang & Turner, 2008; Noir *et al*., 2013; Havko *et al*., 2016). A role for the JAZ‐MYC module in mediating these effects is supported by the observation that *myc2* mutation, as well as overexpression of the JAZ10.3 alternative splice variant, partially inhibits JA‐ and wound‐induced growth stunting (Yan *et al*., 2007; Zhang & Turner, 2008). Our data showing that *mycT* completely rescues the reduced size and biomass of *jazQ* are consistent with this view, as is the finding that *mycT* leaves are larger than Col‐0 leaves under our growth conditions. These collective observations highlight a key role for MYC2/3/4 TFs as negative regulators of leaf growth and biomass accumulation.

The mechanism by which MYC TFs restrict leaf growth remains unknown but several hypotheses can be considered. First, the ability of MYCs to repress the expression of photosynthesis‐associated genes (Yadav *et al*., 2005; this work) suggests that JA‐induced reduction in photosynthetic efficiency may be linked to reduced leaf growth. The photosynthetic robustness of JA‐elicited Arabidopsis leaves, however, does not support this hypothesis (Attaran *et al*., 2014; Campos *et al*., 2016). Second, MYCs may directly repress the activity of positive regulators of leaf cell division or expansion (Pauwels *et al*., 2008; Zhang & Turner, 2008; Noir *et al*., 2013). Such a mechanism would be analogous to the role of MYC2 in repressing the activity of PLETHORA TFs, which promote auxin‐dependent control of cell proliferation specifically in the root stem cell niche (Chen *et al*., 2011). A third possibility is that JA‐induced activation of MYC activity increases the production of GLSs and other defensive compounds whose biosynthesis limits resource allocation to growth (Paul‐Victor *et al*., 2010). Recent studies, however, show that GLS‐based leaf defenses can be expressed in the absence of a growth penalty, indicating that growth–defense antagonism in this and perhaps other genotypes cannot simply be explained by allocation costs (Campos *et al*., 2016; Kliebenstein, 2016; Züst & Agrawal, 2017). In this context, uncoupling of growth–defense tradeoffs in *jazQ phyB* implies that rewiring of phyB−JA crosstalk can override MYC‐mediated growth restriction while leaving MYC‐mediated defenses intact (Campos *et al*., 2016). Although DELLA proteins have been implicated in JA‐mediated growth inhibition of Arabidopsis roots (Hou *et al*., 2010) and hypocotyls (Yang *et al*., 2012), DELLAs are not required for wound‐ and JA‐induced growth stunting of leaves (Zhang & Turner, 2008). Nevertheless, we cannot exclude the possibility that changes in MYC stability (Chico *et al*., 2014), DELLA protein abundance (Leone *et al*., 2014), or direct interaction between DELLA and MYC TFs (Hong *et al*., 2012) contributes to the mechanism by which leaf growth is attenuated by MYC activity. A better understanding of how plants balance leaf growth and defense traits may benefit from research to determine how JA signaling is integrated into regulatory networks that control leaf cell division and expansion (Chitwood & Sinha, 2016; Nelissen *et al*., 2016; Mao *et al*., 2017).

## Author contributions

I.T.M., Y.Y. and G.A.H. designed the research plans; I.T.M., Y.Y., M.L.C., G.K., D.d.O.F., K.S., and X‐F.X. performed the experiments; I.T.M., Y.Y., G.K., K.S., X‐F.X., S.Y.H., and G.A.H. analyzed the data; I.T.M. and G.A.H. wrote the manuscript.

## Supporting information

Please note: Wiley Blackwell are not responsible for the content or functionality of any Supporting Information supplied by the authors. Any queries (other than missing material) should be directed to the *New Phytologist* Central Office.

**Fig. S1** *jazQ mycT* and *myc2345* pedigrees.
**Fig. S2** *sjq10* and *sjq66* carry suppressor mutations in JA biosynthesis and signaling genes.
**Fig. S3** JA responsiveness and rosette growth phenotypes of *mycT* and *myc2345*.
**Fig. S4** Leaf chlorophyll concentrations are comparable among Col‐0, *jazQ, mycT* and *jazQ mycT* in the absence of exogenous JA.
**Fig. S5** Number of genes up‐ and down‐regulated in *jazQ*,* mycT*, and *jazQ mycT* relative to Col‐0.
**Fig. S6** JAZs and MYCs regulate the expression of genes associated with glucosinolate biosynthesis.
**Fig. S7** JAZs regulate the expression of genes associated with glucosinolate hydrolysis in manner independent of MYCs.
**Fig. S8** Increased accumulation of photosynthesis‐associated mRNAs in *mycT*.
**Fig. S9** Loss of MYC2/3/4 increases photosynthetic rate.
**Fig. S10** Transcript levels of *FT* correspond with flowering time in higher order *jazQ* mutants.
**Table S1** Primers for genotyping *jaz* and *myc* mutants

**Table S2** RNAseq analysis performed on WT, *jazQ*,* mycT* and *jazQ mycT* seedlings
