# Supplementary material for: Regulation of growth–defense balance by the JASMONATE ZIM‐DOMAIN (JAZ)‐MYC transcriptional module
Source: New Phytol. 2017 Jun 26;215(4):1533–47. doi: 10.1111/nph.14638 (PMC5542871; doi:10.1111/nph.14638)
Supplement: Supplementary file 1 — Fig. S1 jazQ mycT and myc2345 pedigrees. Fig. S2 sjq10 and sjq66 carry suppressor mutations in JA biosynthesis and signaling genes. Fig. S3 JA responsiveness and rosette growth phenotypes of mycT and myc2345. Fig. S4 Leaf chlorophyll concentrations are comparable among Col‐0, jazQ, mycT and jazQ mycT in the absence of exogenous JA. Fig. S5 Number of genes up‐ and down‐regulated in jazQ, mycT, and jazQ mycT relative to Col‐0. Fig. S6 JAZs and MYCs regulate the expression of genes associated with glucosinolate biosynthesis. Fig. S7 JAZs regulate the expression of genes associated with glucosinolate hydrolysis in manner independent of MYCs. Fig. S8 Increased accumulation of photosynthesis‐associated mRNAs in mycT. Fig. S9 Loss of MYC2/3/4 increases photosynthetic rate. Fig. S10 Transcript levels of FT correspond with flowering time in higher order jazQ mutants. Table S1 Primers for genotyping jaz and myc mutants [file NPH-215-1533-s001.pdf]

New Phytologist **Supporting Information**

Article title: **Regulation of growth-defense balance by the JAZ-MYC transcriptional module**

Authors: Ian T. Major, Yuki Yoshida, Marcelo L. Campos, George Kapali, Xiu-Fang Xin, Koichi Sugimoto, Dalton de Oliveira Ferreira, Sheng Yang He, Gregg A. Howe

Article acceptance date: 27 April 2017

The following Supporting Information is available for this article:

Fig. S1 *jazQ mycT* and *myc2345* pedigrees.

Fig. S2 *sjq10* and *sjq66* carry suppressor mutations in JA biosynthesis and signaling genes.

Fig. S3 JA responsiveness and rosette growth phenotypes of *mycT* and *myc2345*.

Fig. S4 Leaf chlorophyll levels are comparable among Col-0, *jazQ* , *mycT*, and *jazQ mycT* in the absence of exogenous JA.

Fig. S5 Number of genes up- and down-regulated in *jazQ*, *mycT*, and *jazQ mycT* relative to Col-0.

Fig. S6 JAZs and MYCs regulate the expression of genes associated with glucosinolate biosynthesis.

Fig. S7 JAZs regulate the expression of genes associated with glucosinolate hydrolysis in manner independent of MYCs.

Fig. S8 Increased accumulation of photosynthesis-associated mRNAs in *mycT*.

Fig. S9 Loss of MYC2/3/4 increases photosynthetic rate.

Fig. S10 Transcript levels of *FT* correspond with flowering time in higher-order *jazQ* mutants.

Table S1 Primers for genotyping *jaz* and *myc* mutants.

Table S2 RNAseq analysis performed on WT, *jazQ*, *mycT* and *jazQ mycT* seedlings.

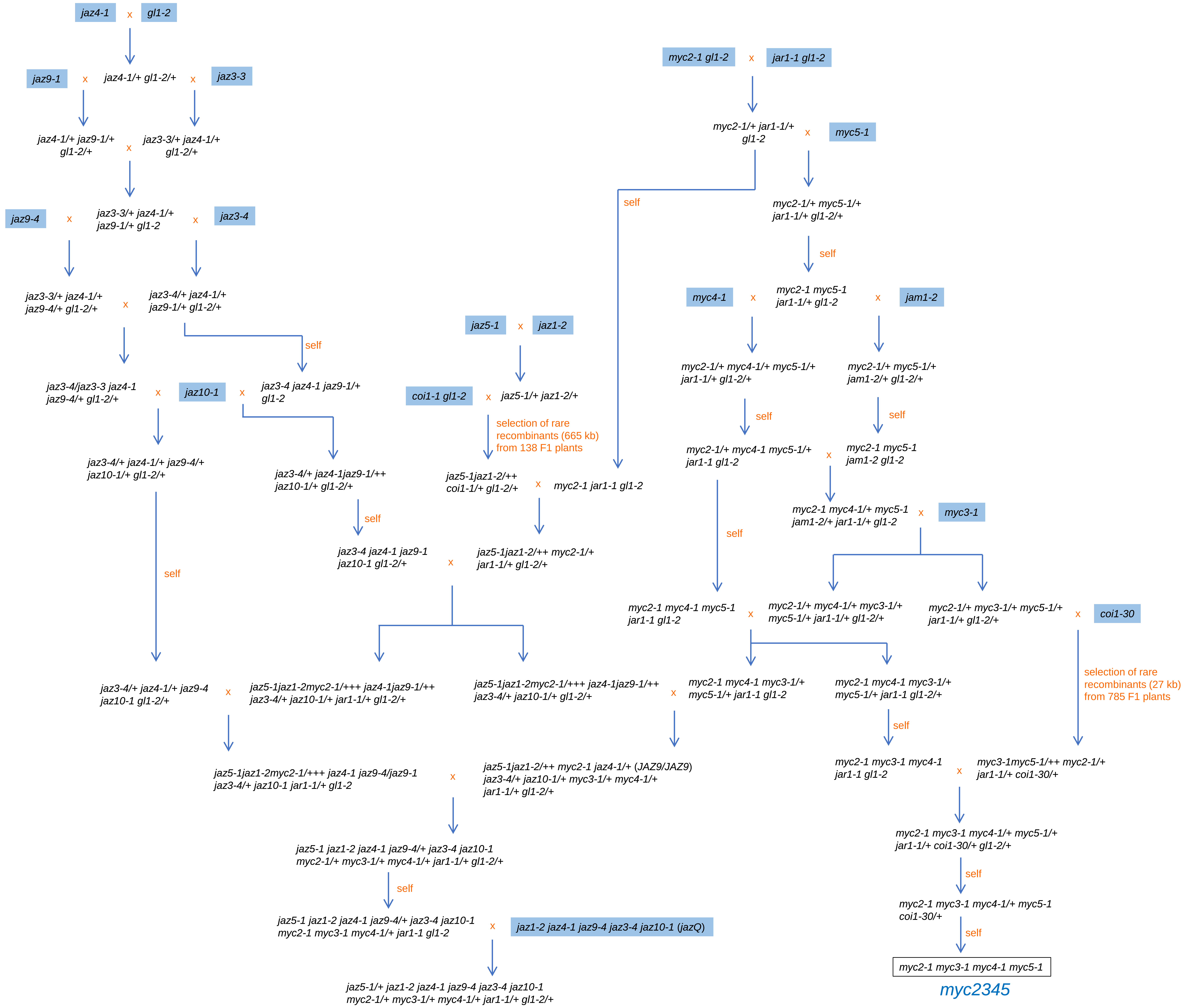

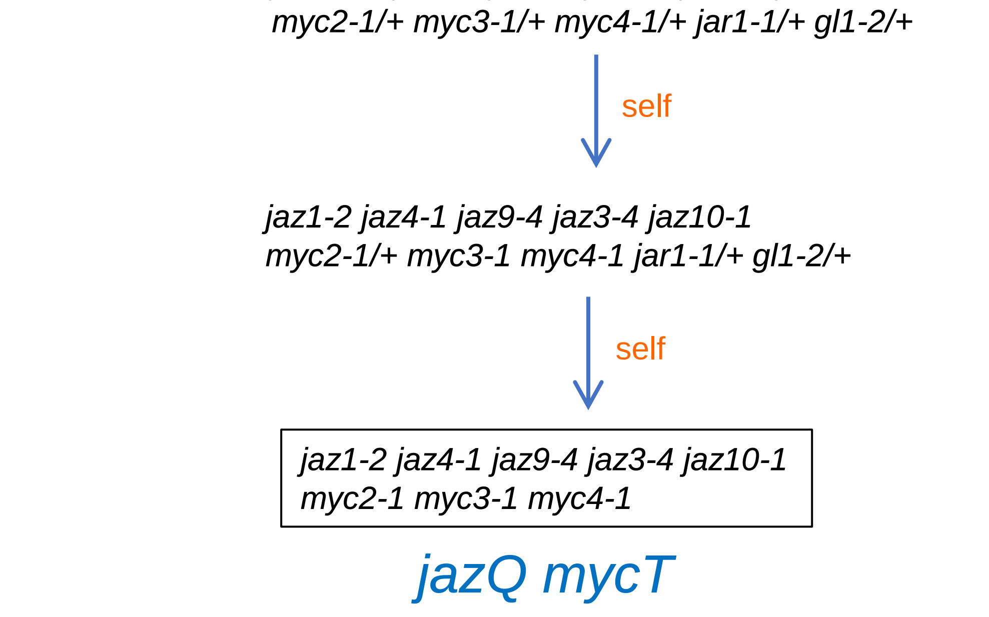

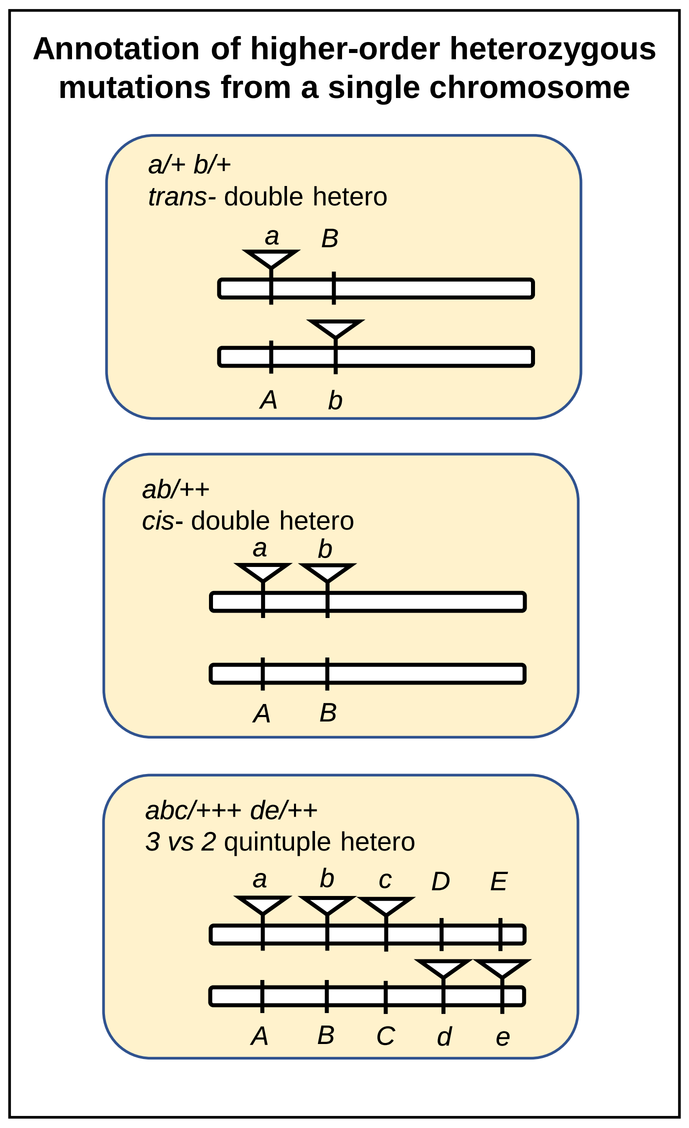

Supplemental Figure S1. *jazQ mycT* and *myc2345* pedigrees.

Red 'x' denotes cross-pollination, whereas red 'self' denotes self-pollination. Blue arrows indicate one generation. Shaded mutants have been reported previously (see references below). Left inset describes the annotation scheme used for higher-order heterozygous mutations from a single chromosome. Our overall breeding strategy was not dedicated for the construction of *jazQ mycT* or *myc2345* but was part of a larger effort to combine mutations in multiple components of the core JA signaling pathway. Due to the limitation of PCR genotyping, the theoretical best genotype was not necessarily obtained in some selection steps. The following mutations were used in this pedigree but ultimately were not included in *jazQ mycT* or *myc2345*: *jaz3-3* and *jaz9-1* were previously characterized (refs.1,2), but *jaz3-4 and jaz9-4* were later selected as alternative alleles; *jaz5-1*, *jar1-1*, and *jam1-2* were initially included but later removed because *JAZ5*, *JAR1*, and *JAM1* were not in the scope of this study; *gl1-2* was included to study trichome development (Yoshida *et al*. unpublished); male sterility of *coi1* mutants was exploited to assist in selection of rare recombination events between closely linked loci (ref.10).

References

1. **Thines *et al*. 2007.** JAZ repressor proteins are targets of the SCF^{COI1} complex during jasmonate signalling. *Nature* **448**: 661-665. (*jaz4-1*, *jaz5-1*, *jaz9-1*); 2. **Chini *et al*. 2007.** The JAZ family of repressors is the missing link in jasmonate signalling. *Nature* **448**: 666-671. (*jaz3-3*); 3. **Yoshida *et al*. 2009.** Jasmonic acid control of GLABRA3 links inducible defense and trichome patterning in *Arabidopsis*. *Development* **136**: 1039-1048. (*gl1-2*, *coi1-1 gl1-2*, *myc2-1 gl1-2*, *jar1-1 gl1-2*); 4. **Sehr *et al*. 2010.** Analysis of secondary growth in the Arabidopsis shoot reveals a positive role of jasmonate signalling in cambium formation. *Plant J* **63**: 811-822. (*jaz10-1*); 5. **Fernandez-Calvo *et al*. 2011.** The *Arabidopsis* bHLH transcription factors MYC3 and MYC4 are targets of JAZ repressors and act additively with MYC2 in the activation of jasmonate responses. *Plant Cell* **23**: 701-715. (*myc3-1*, *myc4-1*); 6. **Yang, Yao, Mei *et al*. 2012.** Plant hormone jasmonate prioritizes defense over growth by interfering with gibberellin signaling cascade. *Proc. Natl. Acad. Sci. USA* **109**: E1192-200. (*coi1-30*); 7. **Sasaki-Sekimoto *et al*. 2013.** Basic helix-loop-helix transcription factors JASMONATE-ASSOCIATED MYC2-LIKE1 (JAM1), JAM2, and JAM3 are negative regulators of jasmonate responses in Arabidopsis. *Plant Physiol* **163**: 291-304. (*jam1-2*); 8. **Figueroa and Browse. 2015.** Male sterility in Arabidopsis induced by overexpression of a MYC5-SRDX chimeric repressor. *Plant J* **81**: 849-860. (*myc5-1*); 9. **Campos, Yoshida, *et al*. 2016.** Rewiring of jasmonate and phytochrome B signalling uncouples plant growth-defense tradeoffs. *Nat Commun* **7**: 12570. (*jaz1-2*, *jaz3-4*, *jaz9-4*, *jazQ*); 10. **Barth and Jander. 2006.** Arabidopsis myrosinases TGG1 and TGG2 have redundant function in glucosinolate breakdown and insect defense. *Plant J* **46**: 549-562.

Supplemental Figure S2. *sjq10* and *sjq66* carry suppressor mutations in JA biosynthesis and signaling genes.
(a,b) Schematic representation of the *ALLENE OXIDE SYNTHASE* (*AOS*) and *CORONATINE INSENSITIVE1* (*COI1*) genes in the suppressor lines *sjq10* (a) and *sjq66* (b). Genomic DNA was extracted from suppressor mutants and used for sequence analysis. Coding exons are shown as black bars, untranslated regions as grey bars, and introns as black lines. The cytosine to thymine transition mutations are illustrated.
(c-h) Photographs of representative five-week old Col-0 (c), *aos* (d), *coi1-30* (e), and *jazQ* (f). Growth phenotypes of *sjq10* and *sjq66* were reconstructed genetically by crossing *aos* and *coi1-1* into the *jazQ* genetic background to generate *jazQ aos* (g) and *jazQ coi1* (h).

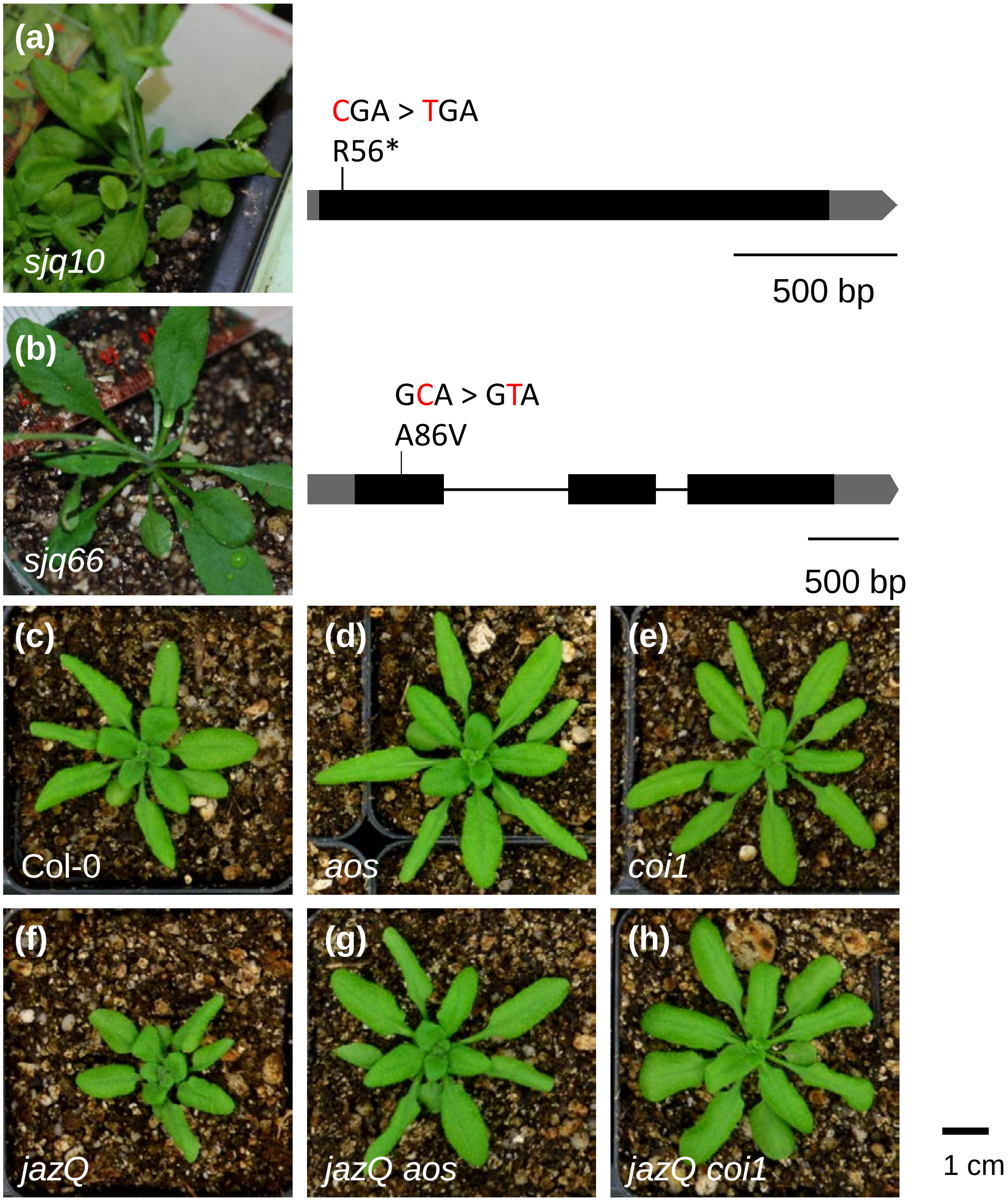

Supplemental Figure S3. JA responsiveness and rosette growth phenotypes of *mycT* and *myc2345*.
(a) Root length of seedlings grown on LS plates supplemented (filled bars) or not supplemented (open bars) with 25 µM MeJA. Bars show the mean ± SD (n = 67-72).
(b,c) Number of leaves (b) and petiole length (c) of three-week-old plants. Bars show the mean ± SD (n = 12).
(d) Anthocyanin content of the entire rosette of 3-week-old plants. Bars denote the mean ± SD (n = 12).
In all grapghs, different letters represent significant differences at P<0.05 determined by two-way ANOVA with Tukey's HSD test. Experiments were repeated 2-3 times with similar results.

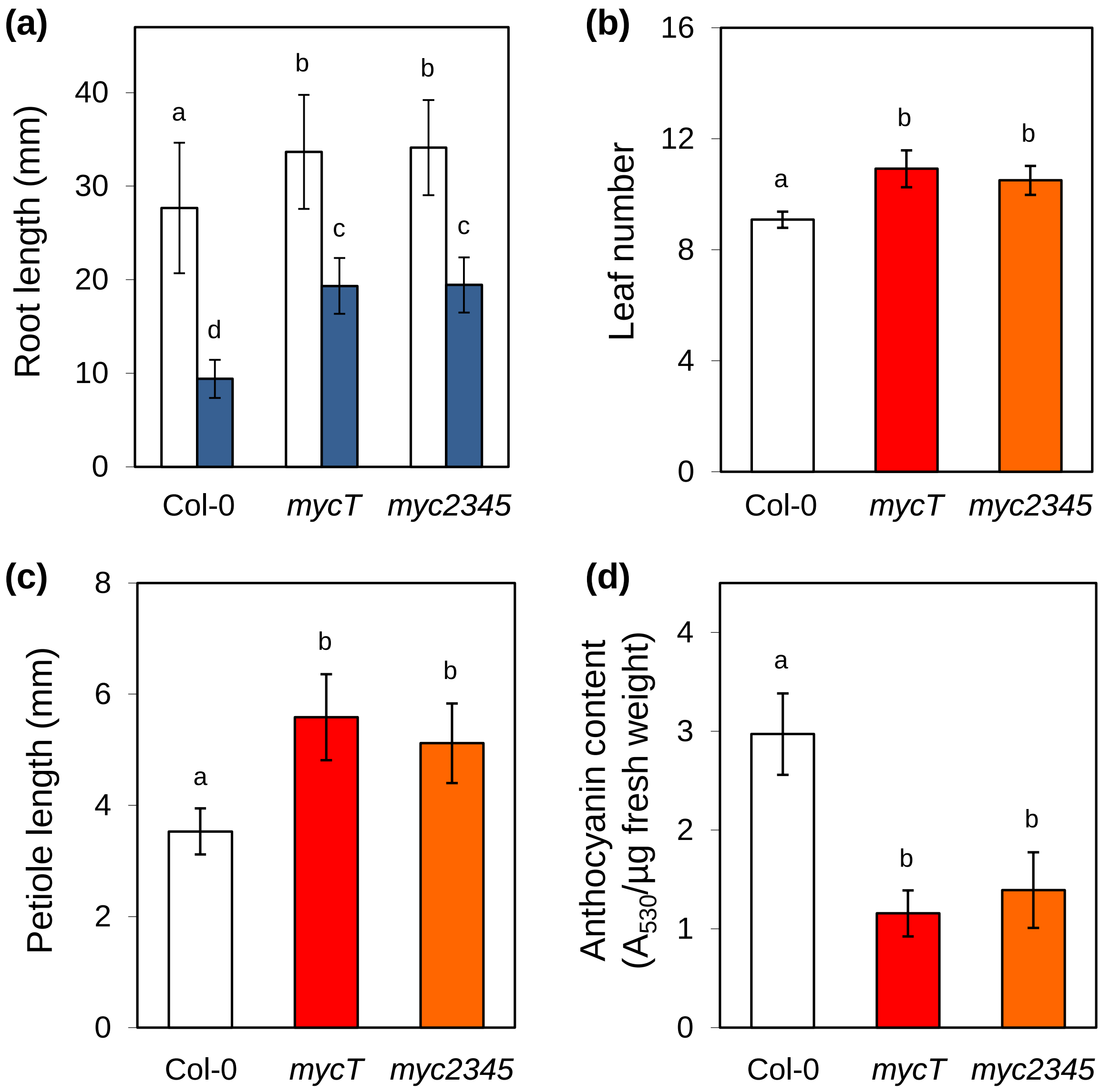

Supplemental Figure S4. Leaf chlorophyll levels are comparable among Col-0, *jazQ* , *mycT*, and *jazQ mycT* in the absence of exogenous JA.
Total leaf chlorophyll in 21-d old soil-grown plants (open bars) or in detached leaves floated on distilled water in the dark for 4 d (black bars). Data are from the same experiment shown in Fig. 2b,c. Bars show the mean ± SD (n = 3 leaves/genotype). Different letters represent significant differences at P<0.05 determined by ANOVA with Tukey's HSD test. Experiment was repeated at three times with similar results.

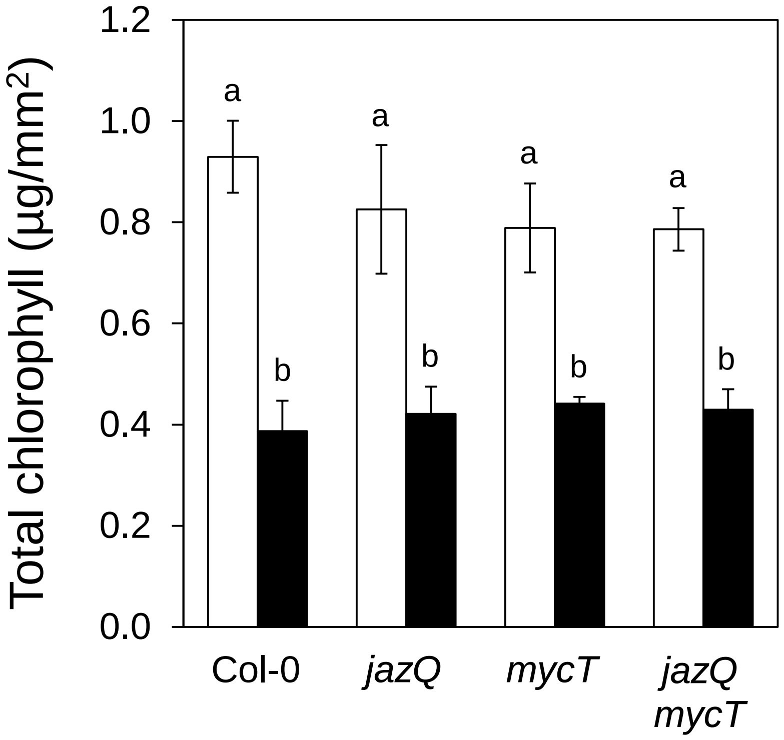

Supplemental Figure S5. Number of genes up- and down-regulated in *jazQ*, *mycT*, and *jazQ mycT* relative to Col-0.

Venn diagrams showing the number of up- regulated (a) and down-regulated (b) genes relative to Col-0 (P < 0.05, determined by DESeq with Benjamini-Hochberg adjustment), and the overlap of genes up-regulated in *jazQ* and *jazQ mycT* seedlings with genes up-regulated in roots of the *ninja1* mutant (c; **Gasperini *et al*. 2015.** Multilayered organization of jasmonate signalling in the regulation of root growth. *PLoS Genet* **11**: e1005300).

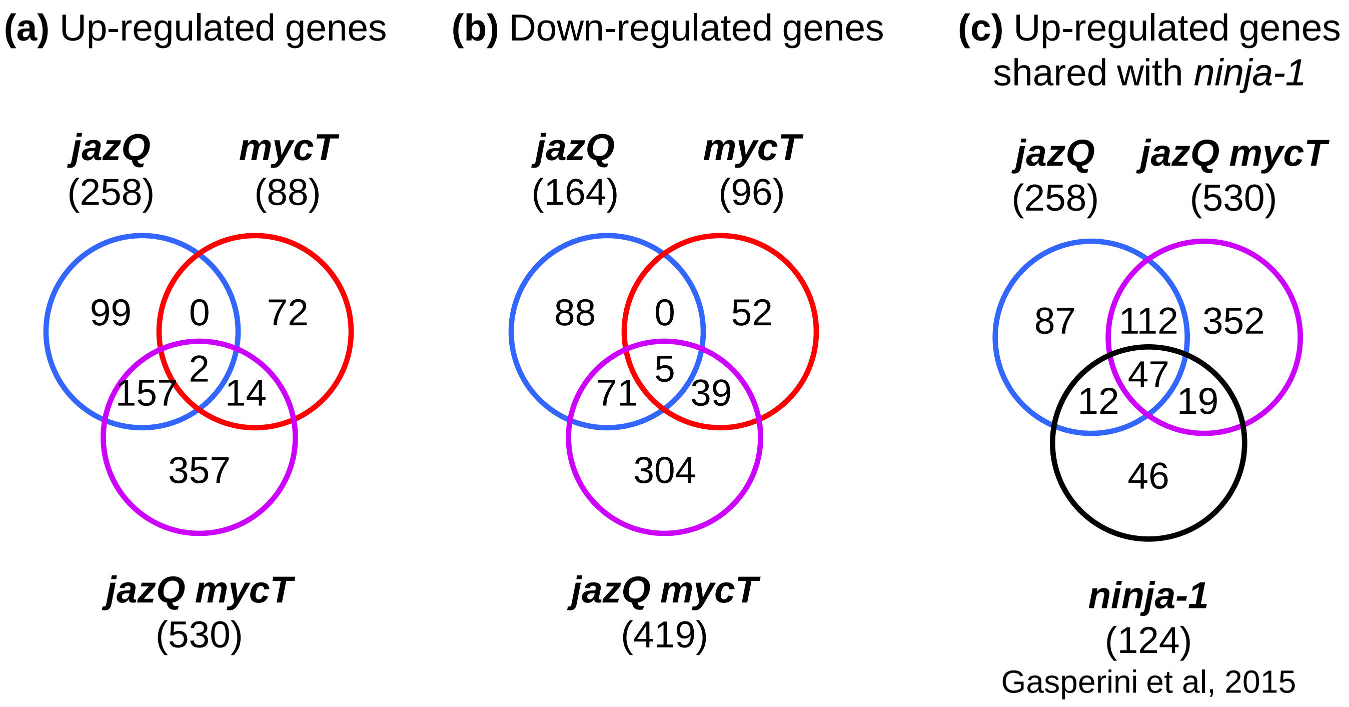

Supplemental Figure S6. JAZs and MYCs regulate the expression of genes associated with glucosinolate biosynthesis.

Biosynthesis pathways for aliphatic (blue) and indolic (violet) glucosinolate biosynthesis (a), with inset showing regulatory MYB transcription factors. RNA-seq expression of corresponding glucosinolate genes in *jazQ*, *mycT*, and *jazQ mycT* relative to Col-0 (b).

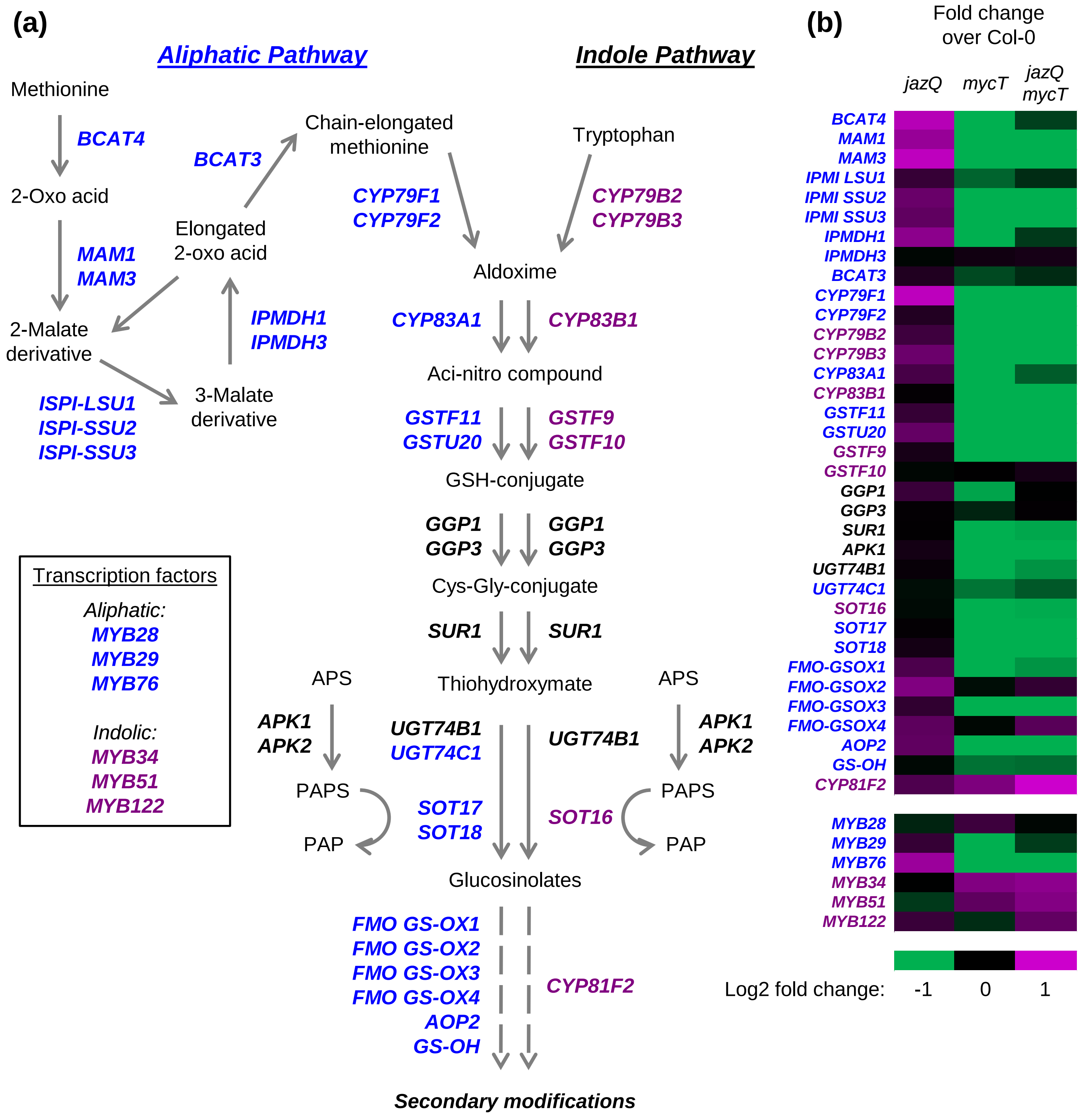

Supplemental Figure S7. JAZs regulate the expression of genes associated with glucosinolate hydrolysis in manner independent of MYCs.
Simplified hydrolysis pathways for glucosinolates (a) with RNAseq expression of corresponding genes in *jazQ*, *mycT*, and *jazQ mycT* relative to Col-0 shown in a heatmap (b) and bar graphs of selected genes (c). Errors bars are standard deviations of the mean of three biological replicates. Expression levels are transcripts per million (TPM). Genes are described in **Wittstock and Burow. 2010.** Glucosinolate breakdown in Arabidopsis: mechanism, regulation and biological significance. *Arabidopsis Book* **8**: e0134.

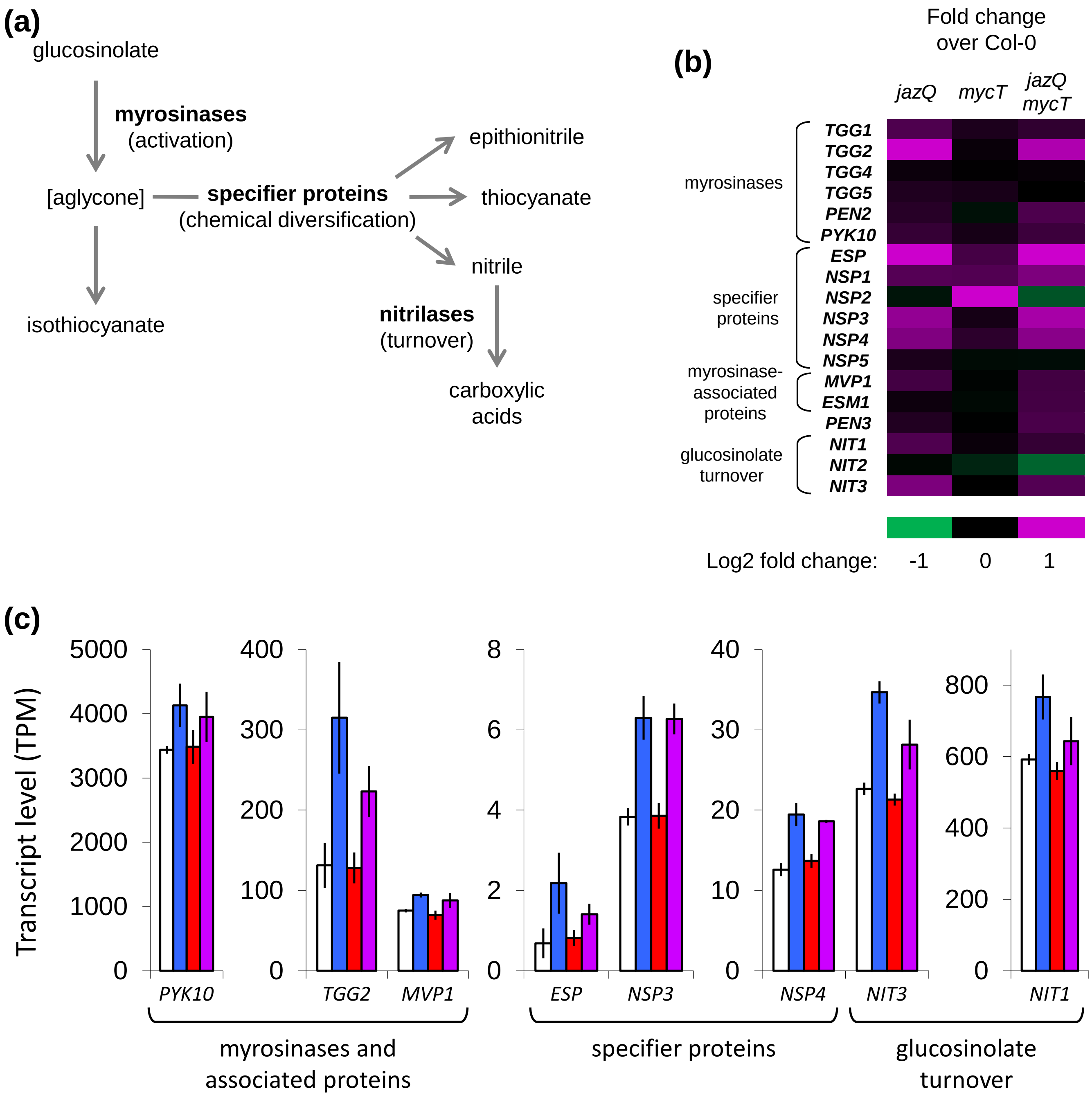

Supplemental Figure S8. Increased accumulation of photosynthesis-associated mRNAs in *mycT*. Schematic representation of photosynthesis-associated genes (a) and corresponding expression in *jazQ*, *mycT*, and *jazQ mycT* relative to Col-0 (b). i, cytochrome b6/f complex; ii, plastocyanin; iii, ferredoxin; iv, ATP synthase; v, CO_2 fixation; vi, reduction reactions; vii, RuBP regeneration; PSI, photosystem I; PSII, photosystem II.

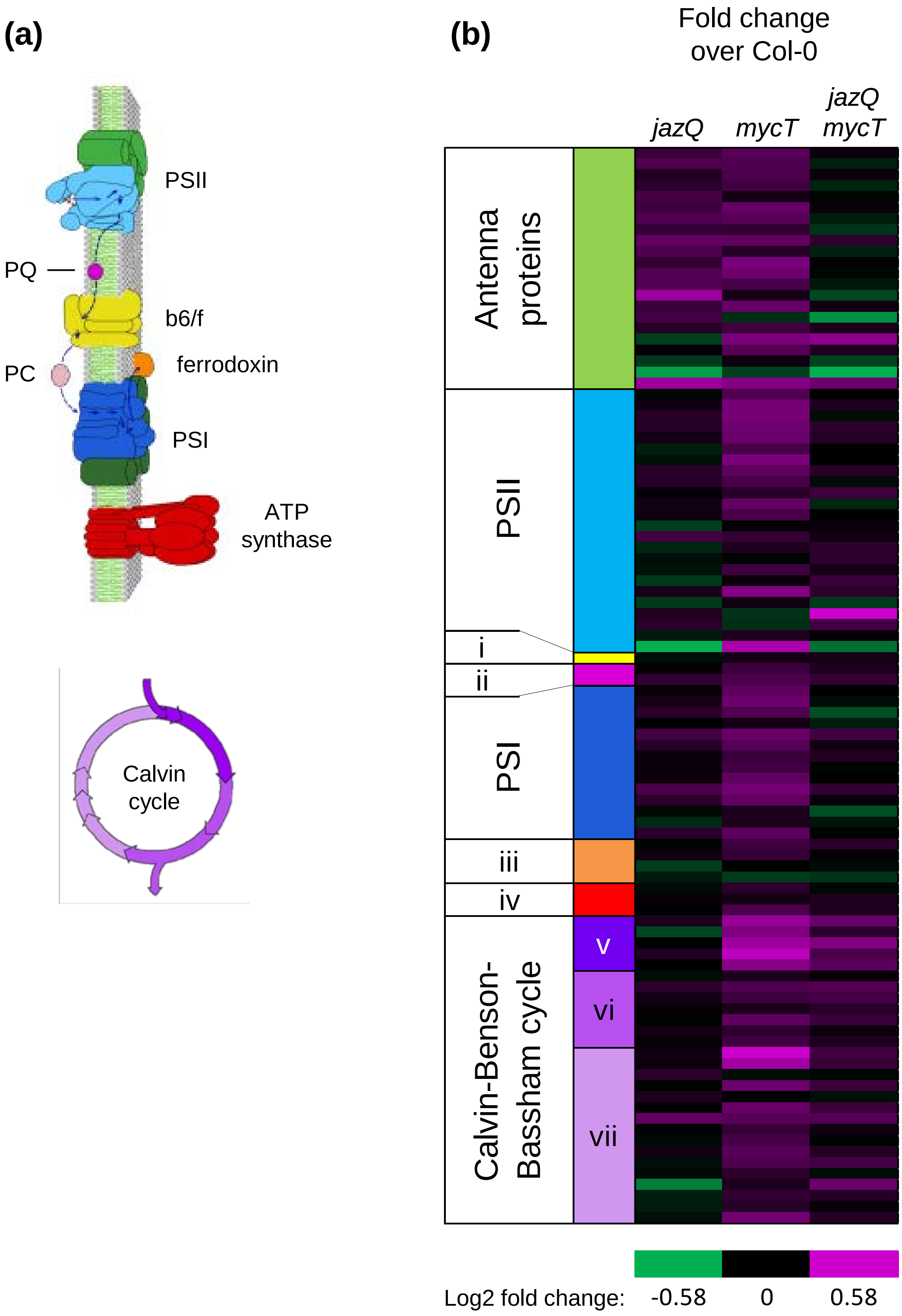

Supplemental Figure S9. Loss of MYC2/3/4 increases photosynthetic rate.
Photosynthetic rates of Col-0, *jazQ*, *mycT*, and *jazQ mycT* plants in response to increasing CO_2 (a) or light (b). Errors bars are standard deviations of the mean of four biological replicates.

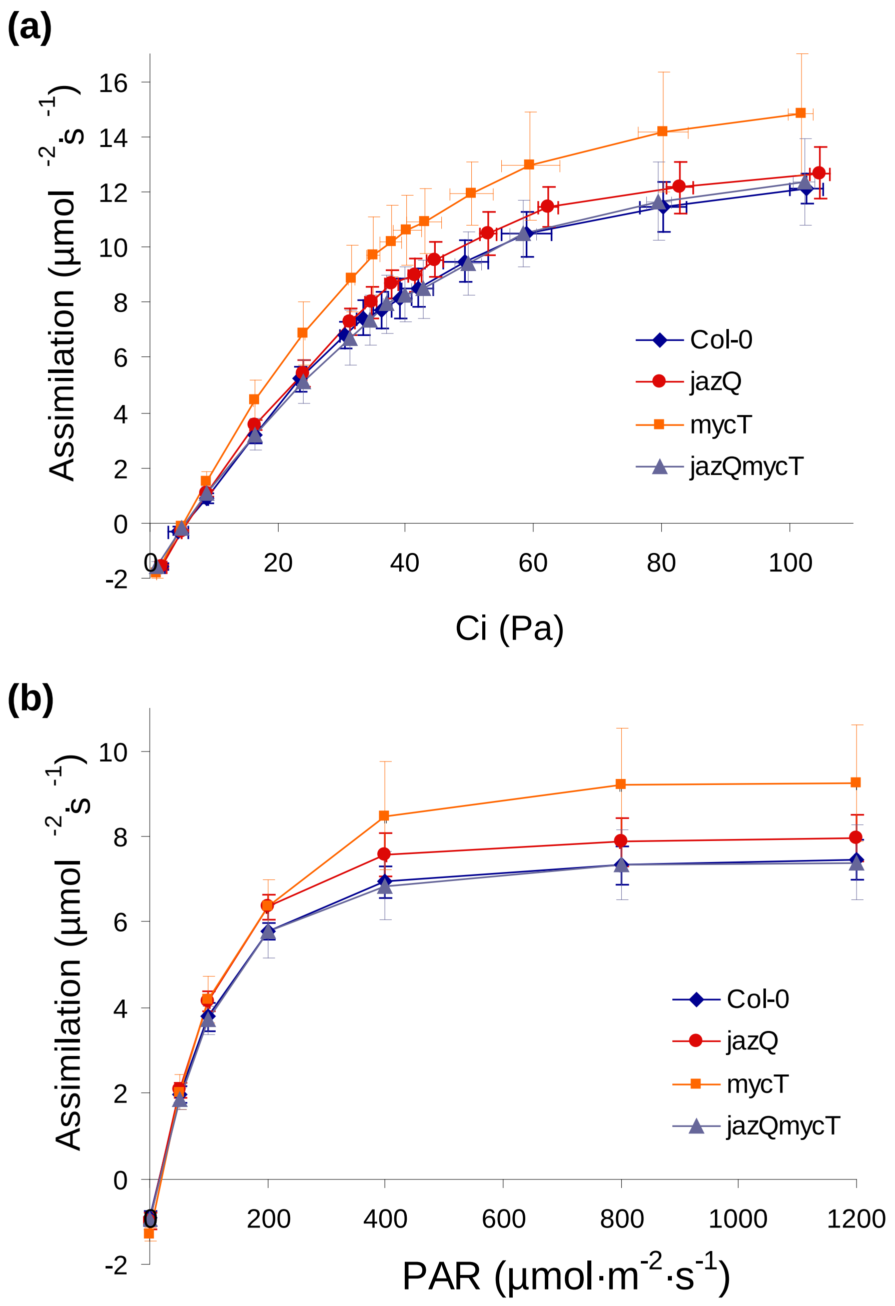

Supplemental Figure S10. Transcript levels of FT correspond with flowering time in higher-order *jazQ* mutants.
Errors bars are standard deviations of the mean of three biological replicates. Asterisks denote statistically different expression levels compared with Col-0 at $P < 0.05$, as determined by DESeq with Benjamini-Hochberg adjustment for multiple testing.

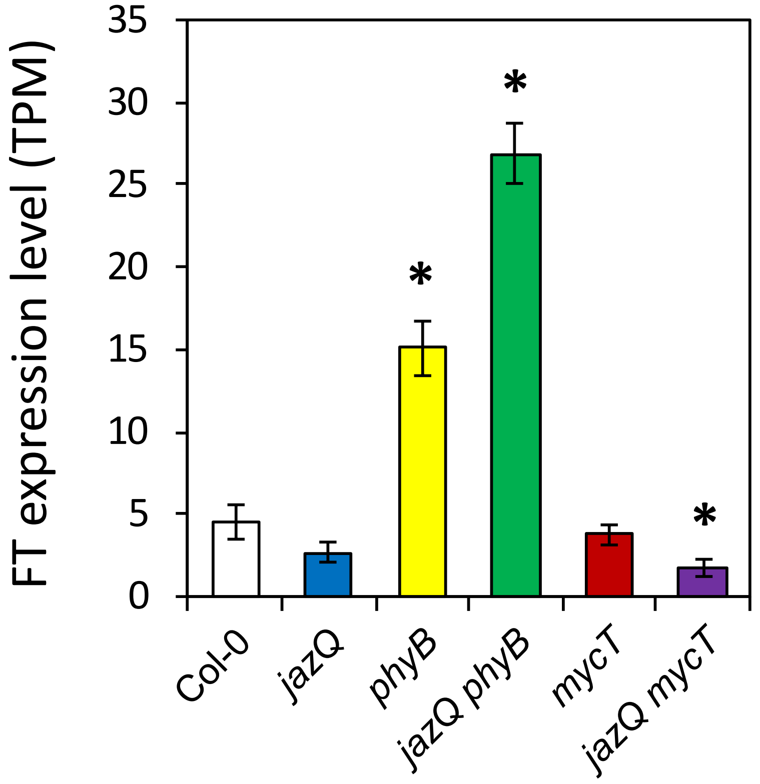

Table S1. Primers for genotyping *jaz* and *myc* mutants.

Gene	AGI accession	Mutant name	Forward primer	Reverse primer	T-DNA border primer
JAZ1	AT1G19180	JIC-SM.22668	ACCGAGACACATTCCCGATT	CATCAGGCTTGCATGCCATT	ACGAATAAGAGCGTCCATTTTAGAG
JAZ3	AT3G17860	GK-097F09	ACGGTTCCTCTATGCCTCAAGTC	GTGGAGTGGTCTAAAGCAACCTTC	ATAACGCTGCGGACATCTACATT
JAZ4	AT1G48500	SALK_141628	TCAGGAAGACAGAGTGTTCCC	TGCGTTTCTCTAAGAACCGAG	TTGGGTGATGGTTCACGTAG
JAZ9	AT1G70700	GK-265H05	TACCGCATAATCATGGTCGTC	TCATGCTCATTGCATTAGTCG	CTTTGAAGACGTGGTTGGAACG
JAZ10	AT5G13220	SAIL_92_D08	ATTTCTCGATCGCCGTCGTAGT	GCCAAAGAGCTTTGGTCTTAGAGTG	GTCTAAGCGTCAATTTGTTTACACC
MYC2	AT1G32640	SALK_040500	GCTACAACCAACGATGAATC	TCATCAACAGCGTCATCCGA	TTGGGTGATGGTTCACGTAG
MYC3	AT5G46760	GK-445B11	GTTAGATCAGCTGCGAATGATTCGG	CTCCGACTTTCGTCATCAAAGCAAC	ATAACGCTGCGGACATCTACATT
MYC4	AT4G17880	GK-491E10	GGATCCATGTCTCCGACGAATGTTCAAGTA	TCTCTCACAACTTGATCCAGCTAA	ATAACGCTGCGGACATCTACATT
